# The roles of APOBEC-mediated RNA editing in SARS-CoV-2 mutations, replication and fitness

**DOI:** 10.1038/s41598-022-19067-x

**Published:** 2022-09-13

**Authors:** Kyumin Kim, Peter Calabrese, Shanshan Wang, Chao Qin, Youliang Rao, Pinghui Feng, Xiaojiang S. Chen

**Affiliations:** 1 Molecular and Computational Biology Section, University of Southern California, Los Angeles, CA 90089 USA; 2 Quantitative and Computational Biology Department, University of Southern California, Los Angeles, CA 90089 USA; 3 Section of Infection and Immunity, Herman Ostrow School of Dentistry, Norris Comprehensive Cancer Center, University of Southern California, Los Angeles, CA 90089 USA; 4 Genetic, Molecular and Cellular Biology Program, Keck School of Medicine, University of Southern California, Los Angeles, CA 90089 USA; 5 Center of Excellence in NanoBiophysics, University of Southern California, Los Angeles, CA 90089 USA; 6 Norris Comprehensive Cancer Center, University of Southern California, Los Angeles, CA 90089 USA

**Keywords:** SARS-CoV-2, Microbiology, Pathogens

## Abstract

During COVID-19 pandemic, mutations of SARS-CoV-2 produce new strains that can be more infectious or evade vaccines. Viral RNA mutations can arise from misincorporation by RNA-polymerases and modification by host factors. Analysis of SARS-CoV-2 sequence from patients showed a strong bias toward C-to-U mutation, suggesting a potential mutational role by host APOBEC cytosine deaminases that possess broad anti-viral activity. We report the first experimental evidence demonstrating that APOBEC3A, APOBEC1, and APOBEC3G can edit on specific sites of SARS-CoV-2 RNA to produce C-to-U mutations. However, SARS-CoV-2 replication and viral progeny production in Caco-2 cells are not inhibited by the expression of these APOBECs. Instead, expression of wild-type APOBEC3 greatly promotes viral replication/propagation, suggesting that SARS-CoV-2 utilizes the APOBEC-mediated mutations for fitness and evolution. Unlike the random mutations, this study suggests the predictability of all possible viral genome mutations by these APOBECs based on the UC/AC motifs and the viral genomic RNA structure.

## Introduction

The causative agent of the COVID-19 pandemic, the severe acute respiratory syndrome coronavirus-2 (SARS-CoV-2), is a member of the enveloped Coronaviridae family that has a single-stranded positive-sense RNA genome^1,2^. Unlike most RNA viruses that exhibit high mutation rates (Ref.^3^ and references therein), SARS-CoV-2 and other coronaviruses have moderate genetic variability because they have built-in proofreading mechanism in their RNA-dependent RNA-polymerase (RdRP) to correct the errors during viral genome replication and transcription^4^.

However, sequencing data of SARS-CoV-2 from patients revealed persistent accumulation of new mutations over the last two years of the Covid19 pandemic, leading to the emergence of new viral strains that can be more transmissible or virulent^5,6^ or evade vaccines^7–9^. The dominant new viral strain is the Omicron BA.5 which is the most antibody immune evasive against currently available vaccines^9^. Therefore the continued emergence of more transmissible and vaccine evasion strains of the SARS-CoV-2 highlights the importance of understanding the driving force of viral mutation and evolution of SARS-CoV-2 genome. There are several possible sources for SARS-CoV-2 viral mutations: spontaneous random errors that are not corrected by the build-in proofreading mechanism of the RdRP, the host-factor mediated mutations of viral genome^3^ (and references therein), and mutations induced by enriched local nucleic acid structure within inverted repeat and epigenetic modification, such as CpG island loci^10^. Recent analysis of SARS-CoV-2 and rubella vaccine virus genome data derived from patients show predominant mutational patterns with specific signatures rather than random genetic variations, suggesting that the host-factor induced mutations play an important role in shaping the viral genomic RNA mutational outcome and evolution^11–14^.

The host responses that can cause mutations on SARS-CoV-2 include reactive oxygen species (ROS) (Ref.^15^ and references therein) and two families of human RNA deaminases: ADARs (the adenosine deaminases acting on RNA) and APOBECs (the apolipoprotein-B (*ApoB*) mRNA editing enzyme, catalytic polypeptide-like proteins)^11,12,14^. The ROS could oxidize nucleic acids to cause viral mutations, which is proposed to be related to the G-to-U and C-to-A mutations^3,16^. The ADAR enzymes modify adenosine to inosine to cause A-to-G mutations in double-stranded RNA (dsRNA), which play important roles in immune regulation (Refs.^17,18^ and references therein).

The APOBEC proteins are a family of cytosine deaminases that can deaminate cytosine to uracil (C-to-U) in single-stranded nucleic acids and function in a variety of biological processes, including innate and adaptive immune responses to viral pathogens (Refs.^19–22^ and references therein). The seven APOBEC3 subfamilies (including A3A to A3H) are reported to restrict DNA and RNA viruses (reviewed in Refs.^21,22^). While most APOBECs use single-stranded DNA (ssDNA) as substrate for C-deamination (reviewed in Ref.^19^), three APOBECs, APOBEC1 (A1)^23,24^, APOBEC3A (A3A)^25^, and APOBEC3G (A3G)^26^, are also shown to deaminate certain cellular single-stranded RNA (ssRNA) targets to cause C-to-U editing. Interestingly, the database analysis of SARS-CoV-2 genomic variations showed an overwhelmingly high C-to-U mutation rate, account for about 40% of all single nucleotide variations, which was interpreted as a result of RNA editing by host APOBECs rather than random mutations^11,12,14,27^. However, there is no report on the direct experimental evidence to demonstrate if APOBECs can edit the SARS-CoV-2 genome and, if so, what is the extent of editing on the viral genome by different APOBECs, and the potential effect of the APOBEC-editing on the virus. In this study, we investigated whether APOBEC proteins can directly edit the RNA sequence of SARS-CoV-2 to generate C-to-U mutations and how such mutations may impact viral replication and viral progeny production in an experimental system.

## Results

### APOBEC-mediated editing test of SARS-CoV-2 RNA

**Figure 1:**
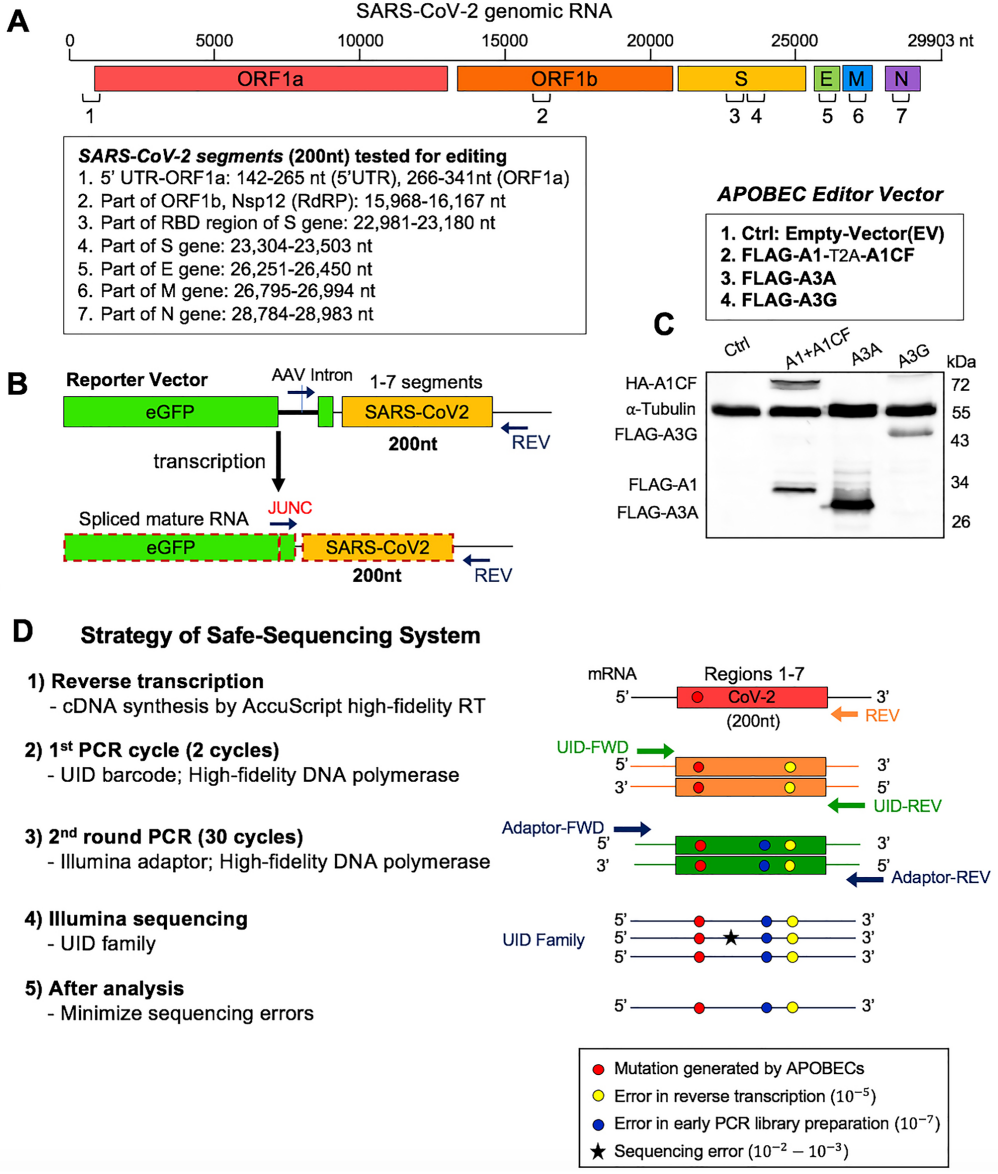
APOBEC-mediated editing test of SARS-CoV-2 RNA. (**A**) Diagram of the SARS-CoV-2 genomic RNA, showing the positions (box) of the seven RNA segments (1–7) selected for studying the RNA editing by APOBECs. (**B**) Reporter vector (top) that contain each of the seven selected viral RNA segments that are transcribed into an RNA containing an AAV intron between the eGFP and the viral RNA segment. Splicing out the AAV intron yields a mature mRNA with a new spliced junction sequence (JUNC) that differs from its coding DNA, which can be used to selectively amplify either the mature mRNA or the coding DNA. (**C**) Three APOBEC editor vectors (top, A1-2A-A1CF, A3A, and A3G) and the Western blot showing their expression in 293 T cells (bottom). A1-2A-A1CF is constructed as one open reading frame (ORF) with a self-cleavage peptide T2A inserted between A1 and A1CF, which produced individual A1 and A1CF proteins in a 1:1 ratio^28,58^. (**D**) Strategy of the Safe-Sequencing-System (SSS) to minimize errors from PCR amplification and sequencing. After the SARS-CoV-2 RNAs from cell extracts are reverse transcribed, the cDNAs are sequentially amplified by the UID barcode (2 cycles) and the Illumina adapter (30 cycles). This SSS approach will distinguish the C-to-U mutations caused by APOBECs from the PCR and sequencing errors (see “Methods”).

Our first goal is to test an experimental system to that can efficiently address whether a particular APOBEC enzyme can actually edit SARS-CoV-2 RNA. We adapted our previously reported cell-based RNA editing system^28^ to examine the ability of APOBEC proteins to edit SARS-CoV-2 genomic RNA to cause C-to-U mutations. Because A1 + A1CF (APOBEC1 Complementation Factor), A3A, and A3G are the three APOBEC proteins shown to possess RNA editing activities^24–26^, we tested each of these three APOBEC proteins for their ability to edit SARS-CoV-2 RNA. Due to technical and budget limitations for the so called “error-free” safe sequencing system (SSS)^29,30^, we selected seven 200 nt-long RNA segments across the SARS-CoV-2 genome for the APOBEC-editing study (Fig. 1A). These seven segments are selected based on a relatively high UC/AC content in a 200 nt window across the entire viral genome (Supplementary Fig. S1A,B), the frequency of polymorphic regions (Supplementary Fig. S1C), and the coverage of various viral genomic areas from the 5′ to the 3′ end, including the 5′-untranslated region (5′UTR) (Fig. 1A). Two regions of the Spike gene were selected due to its pathogenic importance. The selected 200 nt viral RNA segments were constructed into a DNA reporter vector by inserting the corresponding DNA fragments downstream of the eGFP coding sequence. The mRNA transcripts containing eGFP-target RNA will be transcribed by a constitutive promoter on the reporter vector, which enables high-level of eGFP-target RNA transcription to facilitate the detection and quantification of editing on the target RNA (Fig. 1B). An AAV intron is inserted in the middle of eGFP that can be useful to differentiate the mature mRNA transcript (with the intron spliced out) from the coding DNA containing the intron. A primer annealing to the exon-exon junction on the mature mRNA (JUNC, Fig. 1B) can specifically amplify the RNA, but not the coding DNA, by PCR, making it possible to rule out the C-to-U deamination on DNA from the direct C-to-U RNA editing by APOBECs. The reporter vector was co-transfected with the APOBEC editor vector to express the selected APOBEC protein in HEK293T cells (Fig. 1C). Total RNAs were extracted from the cells for cDNA preparation for sequencing using the SSS approach as described below.

To minimize the sequencing errors when evaluating the C-to-U RNA editing, we employed the SSS system, a targeted next generation deep-sequencing system, with slightly adapted protocols (Fig. 1D, see Methods in SI)^29,30^. This SSS method involves the following four critical steps. First, the AccuScript high-fidelity reverse transcriptase (known to have ~${10}^{-4}$–${10}^{-5}$ error rates) was used for the initial reverse transcription of the target SARS-CoV-2 RNA transcripts from the cells to single-stranded cDNA. The JUNC forward primer is used to ensure only mature spliced mRNA segments of SARS-CoV-2 were amplified (Fig. 1B). Second, 2 cycles of initial PCR amplification of the cDNA were performed with a both forward and reverse primers containing a Unique IDentifier (UID), a string of 15 nt randomized sequences, to attach the large family of different UID barcodes (~ ${4}^{30}$), discerning each original target molecule. Third, the initial 2 cycle-PCR products were purified and then amplified with Illumina adaptors (PCR error rate is ~ ${10}^{-7}$). Finally, the high rates of errors from paired-end Illumina sequencing (PE150, ~${10}^{-2}$–${10}^{-3}$error rates) are minimized by eliminating random mutations in the same UID family (see Methods in SI).

Using this SSS approach in this system to check the APOBEC-mediated mutations overcomes the intrinsic high error rate of standard NGS sequencing (PE150, ~${10}^{-2}$–${10}^{-3}$ error rates). The SSS approach also overcomes the limitation of directly analyzing the deposited consensus sequence of patient-derived SARS-CoV-2 RNA sequences, which neglects many of the non-consensus sequence variants that may be introduced by multiple sources, including APOBEC deamination and sequencing errors. However, the SSS approach has its own drawback: the SSS sequencing costs several times more than a regular NGS sequencing. Each SSS sequencing read covers ~ 150–200 nt, making it too expensive for us to cover the entire 30,000 nt RNA genome of SARS-CoV-2. That is the main reason that we selected only seven viral RNA segments for our editing study.

### Sequence motifs near the APOBEC-edited C on SARS-CoV-2 RNA

**Figure 2:**
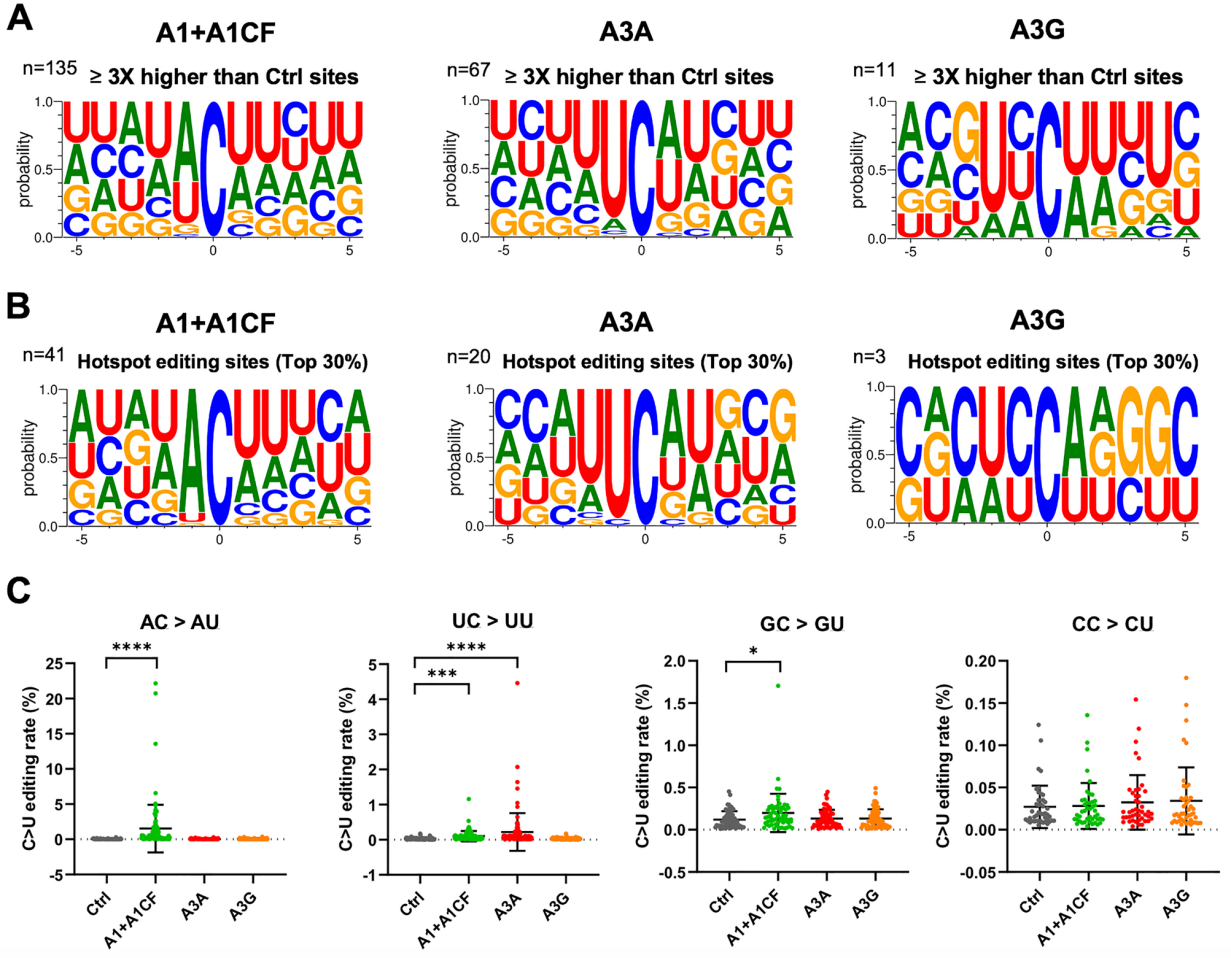
Local sequence context at the APOBEC-edited C sites on SARS-CoV-2 RNA. (**A**) Local sequences around the significantly edited target C sites (± 5 nucleotides from target C at position 0) by A1 + A1CF, A3A, or A3G. The editing level of each C site was normalized to the Ctrl, and only sites with 3 × or higher editing levels than the normalized value were defined as significant editing sites. (**B**) Analysis of local sequences around the top 30% edited C sites (or hotspot editing sites), showing predominantly AC motif for A1 + A1CF, UC for A3A, and CC for A3G. (**C**) Comparison of the C-to-U editing rates (%) of a particular dinucleotide motif by the three APOBECs. Each dot represents the C-to-U editing level obtained from the SSS results. In panel-D, statistical significance was calculated by unpaired two-tailed student’s t-test with *P*-values represented as: P > 0.05 = not significant; not indicated, *P < 0.05, ***P < 0.001, ****P < 0.0001.

In our SSS system to identify the APOBEC-edited C-to-U mutations, the average number of UID families for each of the 28 experiments (seven segments each with three different APOBEC enzymes and one control) has an average of about 130,000 (minimum 85,000, maximum 187,000) from a total of ~ 484 million (paired) reads (Supplementary Dataset File 1). The C-to-U editing levels by each APOBEC are normalized by the control group (Supplementary Dataset File 2). The C-to-U editing by all three APOBECs is detected, with A1 + A1CF and A3A showing much higher editing than A3G (Fig S2). Here, we define the significant target C site where the C-to-U editing efficiency is at least 3 times higher than control. Out of 307 total C in the selected viral RNA segments, the number of significant target sites with A1 + A1CF is 135, A3A is 67, and A3G is 11 (Fig. 2A and Supplementary Dataset File 2). Analysis of the sequence contexts around the significant target C sites (with ± 5 nucleotides from target C) showed that A3A prefers for an UCa/u trinucleotide motif and A1 + A1CF prefers ACu/a motif (Fig. 2A), which is consistent with the reported motif preference for RNA editing by A3A and A1^25,31^. However, A3G did not show a clear motif preference here, possibly due to generally inefficient editing by A3G on a small number of edited sites (n = 11) in the limitedly selected SARS-CoV-2 regions in this study (Fig. 2A). As a control, the sequence context near all C sites (edited + unedited sites) in the selected viral region does not show a specific feature (Supplementary Fig. S3A). The sequence context near the unedited C sites by the corresponding APOBECs also shows no particular trend except for a relatively small ratios change of the − 1 position (e.g., less A by A1 + A1CF and less U by A3A, respectively) (Supplementary Fig. S3B–D).

We also performed the same analysis of the sequence contexts with the top 30% editing efficiency by APOBECs (or hotspot editing sites), which is translated to 38 times higher than control for A1 + A1CF, or 15 times higher than control for A3A, or 6 times higher than control for A3G) (Fig. 2B). It distinctly shows that A3A strongly prefers the UC motif, whereas A1 + A1CF has a strong bias toward the AC motif. However, the preferred motif by A3G is not a clear cut from our data, possibly due to the low activity on the limited number of edited sites among the seven tested viral segments. These results suggest that the observed AC-to-AU mutations are most likely generated by A1 plus cofactor A1CF (or other A1 cofactors, such as RBM47^32^); the UC-to-UU mutations by A3A in the sequence variation detected on the SARS-CoV-2 RNA segments (Fig. 2C). Interestingly, among all the C-to-U variations of the SARS-CoV-2 sequences in our analysis, AC-to-AU (preferred by A1) and UC-to-UU (preferred by A3A) account for 38.23% and 31.83%, respectively, significantly higher than GC-to-GU and CC-to-CU that account for 15.44% and 14.50%, respectively (Supplementary Fig. S4), indicating the significance of APOBEC editing on the viral mutation.

### Features of the efficiently APOBEC-edited RNA sites on SARS-CoV-2

Although each of the three APOBEC proteins showed a strong preference for specific dinucleotide sequence motifs (i.e. AC, UC, or CC sequence motifs) for editing on the viral RNA, the relative editing efficiency of these motif sites vary greatly, such as between 0.0041 and 22.15% for A1 + A1CF, and between 0.0040 and 4.46% for A3A (Supplementary Dataset File 2). Furthermore, many of these AC, UC, and CC motif sites on the viral RNA have no detectable editing by A1 + A1CF, A3A, and A3G, respectively, suggesting that other RNA features beyond the dinucleotide sequence motifs, such as the secondary and tertiary structures, must play a role in the editing efficiency of a particular motif site.

**Figure 3:**
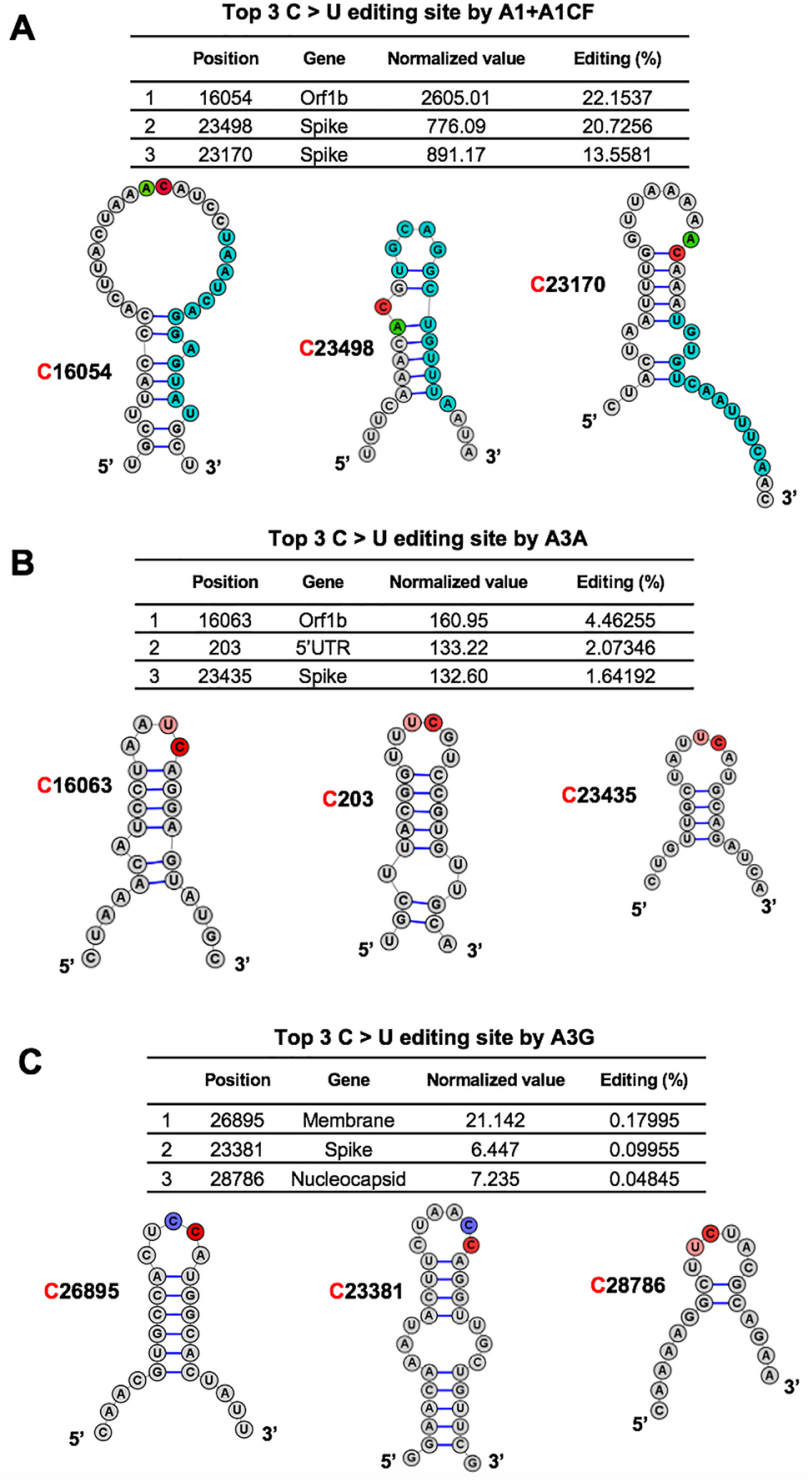
Overall features of the RNA around the most preferred APOBEC-edited sites on SARS-CoV-2. The predicted RNA secondary structures^62^ of the sequences near the top 3 highest editing C sites by A1 + A1CF (**A**), A3A (**B**), and A3G (**C**) (see related Supplementary Dataset File 2). The editing efficiency of each site is listed at the top of each panel. In the secondary structure, the target C sites are highlighted in red, and -1 positions of the target C sites are highlighted in green for A, pink for U, and blue for C, respectively. In panel (**A**), the proposed canonical mooring sequences for A1 + A1CF (highlighted in sky blue) contain relatively high U/A/G contents downstream of the target C.

The RNA editing by A1 + A1CF was previously reported to require a so-called mooring sequence that has a general stem-loop structure around the target C and contains relatively high U/G/A content downstream of the target C^33,34^. However, this requirement for the mooring sequence and stem-loop structures are shown to be quite relaxed and still needs further characterization^28,35^. We analyzed the RNA features around the top 3 AC sites with the highest editing efficiency by A1 + A1CF (Fig. 3A). The result showed that they could form a relatively stable stem-loop structure, with relatively high U/G/A contents downstream of the target C (Fig. 3A). Among these top 3 editing sites, editing at C16054 is significantly higher than at C23170 (Fig. 3A), suggesting that, in addition to the possible involvement of long range RNA interactions, the editing efficiency also depends on the detailed local stem-loop structure and the position of the target C.

Sharma and colleagues recently discovered RNA editing activities by A3A and A3G in human transcripts through RNAseq using the common NGS without the safe sequencing SSS approach^25,26,36^. More than half of target cellular RNA substrates have a stem-loop secondary structure, and the target C locates in the loop region. Interestingly, our top 3 highest A3A-mediated editing sites on SARS-CoV-2 RNA reported here all have the UC motifs in the loop of a predicted stem-loop secondary structure (Fig. 3B). Again, the editing efficiency at C16063 (4.5%) is about threefold higher than the third efficiently edited site at C23453 (1.6%) (Fig. 3B). Analysis of the top 3 sites edited by A3G also showed the CC (or UC) motifs in the single-stranded loop region of a predicted stem-loop secondary structure (Fig. 3C).

To rule out the possibility that the RNA C-to-U mutations result from DNA C-to-U deamination instead of the direct RNA editing by APOBECs, we performed a side-by-side sequencing of the DNA on the reporter vector and its corresponding RNA transcript containing the Orf1b region of SARS-CoV-2 (15,968–16,167 nt). The reporter DNA and the RNA transcript extracted from the cells expressing APOBECs were PCR amplified using the forward prime annealing to either the AAV-intron specific for the DNA or to the JUNC specific for the spliced RNA only. The PCR products from the DNA and mRNA were subjected to Sanger sequencing, and the C-to-U changes were analyzed. No DNA C-to-U mutation was detected, but specific RNA C-to-U changes on the mRNA transcript were present in a frequency consistent with our SSS results obtained in the presence of A1 + A1CF (e.g., C16049, C16054, and C16092, etc.) or A3A (C16063) (Supplementary Fig. S5). These data indicate that the C-to-U changes on the RNA are not caused by DNA mutation, but are the result of direct RNA editing mediated by A1 + A1CF and A3A in our cell-based assay system.

### The potential effect of APOBEC-mediated RNA editing on SARS-CoV-2 variants

**Figure 4:**
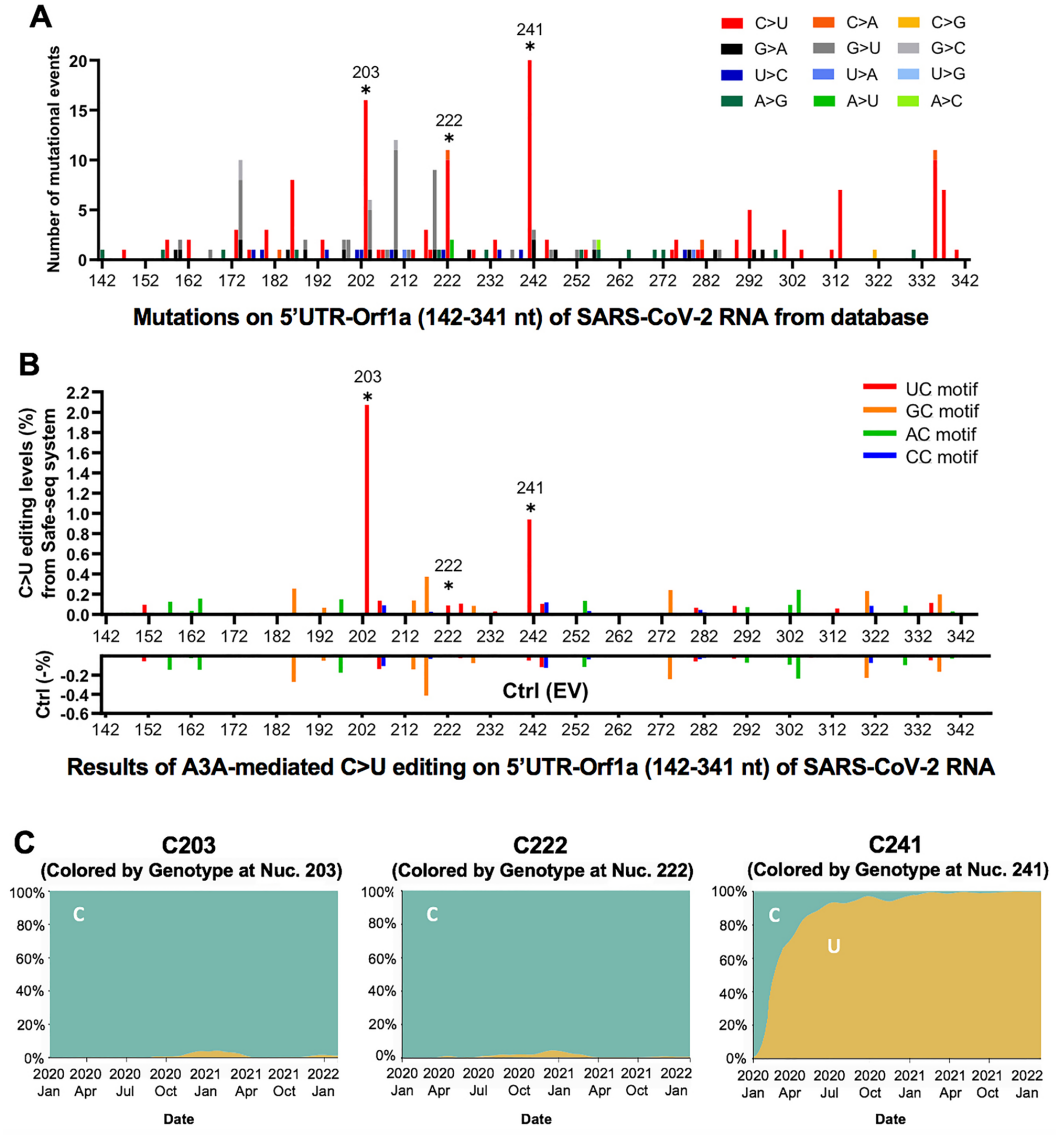
The potential effect of APOBEC-mediated editing on SARS-CoV-2 mutations and fitness. (**A**) The number of mutational events (all single nucleotide variants) on SARS-CoV-2 RNA segment 5’UTR-Orf1a (segment 1 in Fig. 1A) from the SARS-CoV-2 genome sequence data (the Nextstrain datasets from Dec. 2019 to Jan. 22nd, 2022 downloaded from the GISAID database, https://www.gisaid.org/hcov19-variants/ and https://nextstrain.org/ncov/global). The C203, C222, and C241 represent many of the C-to-U mutational events (asterisks) with the A3A-editing UC motif in the SARS-CoV-2 variants. (**B**) The A3A-mediated C-to-U editing rate on UC motif in the same 5′UTR-Orf1a region obtained from our cell-based editing system and the SSS analysis. The Ctrl (EV) editing levels (or background error rates) of the corresponding region are presented as negative values (%). The C203, C222, and C241 (asterisks) all showed significant editing by A3A. (**C**) The C-to-U mutation prevalence over time at C203, C222, and C241. The sequencing frequency is represented by C in blue and U in yellow (referred to the Nextstrain datasets: https://nextstrain.org/ncov/global). This analysis showed that SARS-CoV-2 started to acquire the C-to-U mutation at C241 in January 2020. By July 2020, 90% of the circulating viral variants carry this mutation at C241. By March 2021, almost all circulating viral variants have this mutation, suggesting the C241 to U mutation in the 5’UTR is beneficial to the viral fitness (see Supplementary Fig. S14).

The currently deposited patient-derived SARS-CoV-2 genome sequence databases reflect the selected consensus viral sequences (including some mutations caused by APOBEC-mediated mutations) that survived the selection pressure for fitness. As a result, many of the mutations that are not beneficial or detrimental to the virus may be transient or selected against and won’t be represented in the final consensus sequence or are difficult to be analyzed due to the high sequencing errors of standard NGS sequencing errors. With the direct experimental evidence described here that APOBECs can target specific sites on SARS-CoV-2 for editing, we analyzed the publicly available SARS-CoV-2 genome sequence data (the Nextstrain datasets from Dec. 2019 to Jan. 22nd, 2022 downloaded from the GISIAD database, https://nextstrain.org/ncov/global)^37^, with hope to detect some obvious effects of APOBEC-mediated viral mutations on the current viral strain variants and fitness. The analysis revealed that the C-to-U is the predominant mutation for the entire genome, accounting for ~ 55.8% among all single nucleotide variants (SNVs) within the SARS-CoV-2 5′UTR-Orf1a region (142–341 nt, the segment tested in our reporter 1 vector) (Fig. 4A and Supplementary Fig. S6A). Of particular interest is the prominent mutation occurring at UC203, UC222, and UC241 in the 5′UTR region of these virus variants (Fig. 4A), as these 3 sites all feature UC motifs and showed significant C-to-U editing by A3A in our assay results (Fig. 4B, Supplementary Fig S6B, and Supplementary Dataset File 2). These results suggest that A3A generated these mutations on the viral RNA genome, and the mutations can be maintained, likely because these three mutations generated by A3A editing are not determinantal to the virus. Two of these three mutations, UC203 and UC222, were detected in some of the SARS-CoV-2 sequences in late 2020 but are not persistently present in the main circulating strains, suggesting these two mutations may be neutral for the viral fitness. Surprisingly, the C-to-U mutation at UC241 occurred in early January 2020 and has rapidly become a signature of the dominant strains (including Delta and Omicron) that spread worldwide (Fig. 4C and Supplementary Fig S6C), strongly suggesting that this C-to-U mutation at UC241 may contribute to the better fitness for SARS-CoV-2. Although UC241to U mutation is within 5′UTR, the correlation of this mutation with the dominant new strains is reminiscent of that of the D614G mutation of the spike protein-coding region^5,38^. Because 5′UTR has an important regulatory function for the replication of SARS-CoV-2 RNAs and for the expression of viral proteins^39,40^, the UC241 mutation may affect one or several aspects of these important functions of the 5′UTR in the viral infection steps relating to viral RNA replication, transcription, and translation.

In all representative clades of SARS-CoV-2 emerged over the last two years since the initial outbreak, the C-to-U mutations have been much more pronounced than other types of single nucleotide variations (Supplementary Fig. S7A). Even the very recent omicron variants, which began to spread from Nov. 2021 rapidly, continue to show noticeable C-to-U editing pattern (Supplementary Fig. S7B). Notably, the AC to AU mutation at C23525 resulted an H655Y mutation on the spike protein (Supplementary Fig. S7B), and the H655Y mutation was shown to alter cell entry pathways, i.e. the mutation is responsible for the preferential usage of endosomal pathway over cell surface entry pathways^41^. This result suggests that further studies are necessary to have a comprehensive understanding of the potential effect of APOBEC-mediated C-to-U RNA editing on SARS-CoV-2 mutations and evolution.

### SARS-CoV-2 replication and progeny yield in cells overexpressing APOBECs

**Figure 5:**
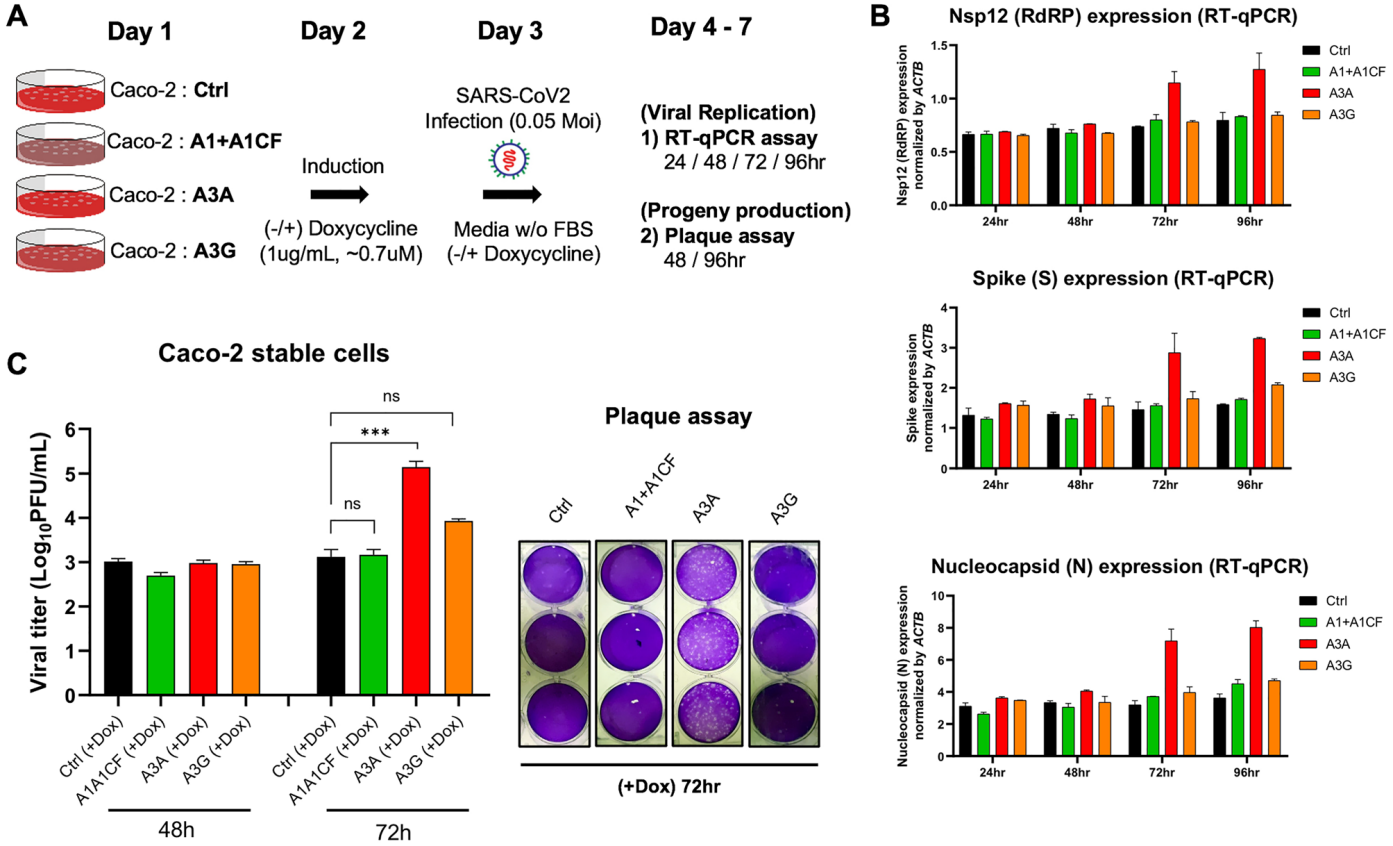
SARS-CoV-2 replication and virion production in cells expressing APOBECs. (**A**) Overview of experiments for SARS-CoV-2 replication and viral production in the presence of APOBECs. The Caco-2 stable cell lines were constructed to express A1 + A1CF, A3A, or A3G under a tetracycline-controlled promoter. The Caco-2-APOBEC stable cell lines were then infected with SARS-CoV-2 (MOI = 0.05), and the viral RNA replication and progeny production were measured at different time points. (**B**) Effect of each APOBEC expression on SARS-CoV-2 viral RNA replication. Measurement of relative viral RNA abundance at different time points after viral infection of the Caco-2-APOBEC stable cell lines expressing A1 + A1CF, A3A, or A3G. The viral RNA abundance was measured using real-time quantitative PCR (qPCR) to detect RNA levels by using specific primers to amplify three separate viral regions, the *Nsp12*, *S,* or *N* coding regions (see Methods in SI). (**C**) Effect of each APOBEC expression on SARS-CoV-2 progeny production. Infectious viral progeny yield harvested in the medium at 48 h and 72 h post-infection was determined by plaque assay (see “Methods”). In panel (**B**) and (**C**), statistical significance was calculated by unpaired two-tailed student’s t-test with *P*-values represented as: P > 0.05 = not significant, ***P < 0.001.

To examine if the three APOBEC proteins can affect SARS-CoV-2 replication and progeny yield in a well-controlled experimental setting, we used the human colon epithelial cell line Caco-2 that expresses ACE2 receptor and thus is a model cell line for SARS-CoV-2 infection and replication studies^42^. Because Caco-2 cell lines have no detectible endogenous expression of A1, A3A, and A3G protein (Supplementary Fig. S8), we first constructed Caco-2 stable cell lines expressing one of the three APOBEC genes. The externally inserted APOBECs are under tetracycline-controlled promoter so that their expression can be induced by doxycycline (Fig. 5A and Supplementary Fig. S9A). The Caco-2-APOBEC stable cell lines were then infected by SARS-CoV-2, and the viral RNA replication and progeny yield were measured and compared with the control cell line without APOBEC expression. The viral RNA abundance as an indicator for RNA replication was measured using real-time quantitative PCR (qPCR) to detect the RNA levels using primers specific for amplifying three viral regions: the Nsp12 region, the S region, and the N region, covering the genomic and subgenomic regions. The viral progeny yield was assayed through plaque assay in Vero E6 cell line using the virions produced from the Caco-2-APOBEC stable cell lines at different time points post-infection (Fig. 5A). Vero E6 cell line is highly sensitive to viral infection because of its defective innate immunity, allowing sensitive quantification of viral progeny produced from the Caco-2 cell lines.

The replication assay results showed that, compared with the control Caco-2 cell line, no significant change of viral RNA level was detected by qPCR with the cell lines expressing A1 + A1CF or A3G even up to 96 h post-infection (Fig. 5B). Unexpectedly, the abundance of viral RNAs has increased significantly in the cell lines expressing A3A at 72 h and 96 h post SARS-CoV-2 infection. These results suggest that, despite the general viral restriction function of APOBECs, presence of A3A expression appears to endow an advantage for the viral RNA replication.

Consistent with the increased viral RNA replication, A3A expression in the stable Caco-2 cell line also correlates with significantly higher viral progeny yield after 3 days of infection. While no difference in viral titer was observed at 48 h from all cell lines with or without APOBEC expression, the virus titer from the cell line expressing A3A consistently showed an approximately 10–100 fold higher than the control and A1 + A1CF and A3G expressing cell lines at 72 h (Fig. 5C). Based on the intriguing results of A3A-related enhancement of SARS-CoV-2 replication and progeny virus yield, we further investigated whether such effects are dependent on the deaminase activity of A3A. We constructed an A3A-knockout (∆A3A) and an A3A catalytically inactive mutant-expressing (A3A-E72A^43,44^) Caco-2 cell lines, and the SARS-CoV-2 replication and progeny yield were compared in the four different Caco-2 cell lines, i.e. original Caco-2 cell line (control), and Caco-2 with A3A-knockout (∆A3A) to make another control cell line even if no A3A mRNA expression can be detected in Caco-2 cells (Supplementary Fig. S8), and the stable cell lines expressing A3A WT or the inactive mutant A3A-E72A (Supplementary Fig. S9B). Again, the abundance of viral RNA based on the qPCR results of the three viral regions (*Nsp12*, *S*, or *N* coding regions) all displayed significant increases in the cell lines expressing A3A-WT at 72 and 92 h post SARS-CoV-2 infection but showed no significant difference in the control Coca-2 cells and the ∆A3A cells (Supplementary Fig. S10A). Furthermore, the viral progeny yield harvested from the A3A-WT expressing cell line also was significantly higher than those from the control Caco-2 cells and the ∆A3A cells (Supplementary Fig. S10B). Interestingly, A3A-E72A also showed slight enhancement of viral RNA replication (Supplementary Fig. S10A) and viral progeny yield (Supplementary Fig. S10B). This low level of deaminase-independent enhancement of viral replication by A3A-E72A inactive mutant would be worth further validation and investigation.

To confirm whether A3A-induced mutations can be observed during SARS-CoV-2 infection in Caco-2 cells, we checked the viral sequences around the C241 on the 5′UTR of the viral genome with standard Sanger sequencing at different time points after viral infection, as shown in Fig. 5A. We reasoned that the C241U mutation should occur and be detectible after 72 h post infection without using the SSS sequencing method if this C241U mutation contributes to the increased viral replication and viral progeny. The results demonstrated that the C241 started to show clearly detectible (and statistically significant) C-to-U mutation 72 and 96 h post infection in Caco-2 cells with WT A3A (Supplementary Fig. S11A,B). This significant C241U mutation correlates with the time points (72 and 96 h) with detectible increases in viral RNA replication and viral progeny production. Contrary to the editing at C241, our sequencing results showed that the nearby C203 and C222, where C203U and C222U mutations displayed a neutral effect on viral replication, showed a slight but statistically non-significant increase in C to U mutations 72 h post viral infection in WT A3A overexpression (Supplementary Fig. S11B). We also checked the potential editing at C16054 and C23535 (AC motif) by A1 + A1CF on the viral genome from the Caco-2 cells infected by SARS-CoV-2 using Sanger sequencing. The results showed a slight but statistically non-significant increase in editing at C16054 and C23535 96 h post viral infection (Supplementary Fig. S12A,B). In summary, while APOBEC-mediated C to U mutations were detected in all five sites on the viral RNA from infected Caco-2 cells using Sanger sequencing, significant C to U mutation was detected only for C241U at 72 and 96 h post infection. This observation suggests that the C241U mutation may be a beneficial mutation for viral replication and progeny production even in cell culture infection assay.

While the editing of SARS-CoV-2 RNA by A3A and A1 + A1CF has been demonstrated in our cell culture system, the analysis of the expression profiles reveals that A3A and A1 + A1CF, but not A3G, are expressed in the human organs and cell types infected by SARS-CoV-2 (Supplementary Fig. S13A,B). Such expression profiles make it possible for A3A and A1 + A1CF to edit the viral RNA genome in the real world. Many human cell types expressing ACE2 in multiple organs can be infected by SARS-CoV-2, including (but not limited to) the lungs, heart, small intestine, and liver^45,46^. A3A is expressed in lung epithelial cells, and, importantly, the A3A expression level is significantly stimulated by SARS-CoV-2 infection in patients^47–49^ (Supplementary Fig. S13A). A1 and its two known cofactors, A1CF and RBM47, are not expressed in the lungs but are expressed in the small intestine or liver^50^ that can also be infected by SARS-CoV-2 (Supplementary Fig. S13B).

Taken together, our results suggest that the A3A deaminase activity plays a major role for promoting the viral replication and viral progeny production. This is consistent, among other things, with the observation that the UC241 to UU241 mutation, a site highly edited by A3A in our study, is within the viral packaging signal and near the viral replication regulation area at the 5′UTR (Supplementary Fig. S14), which could explain why this mutation becomes prevalent in the widely circulating SARS-CoV-2 strains after January 2020 (Fig. 4A–C).

## Discussion

Several new viral strains have emerged in this ongoing COVID19 pandemic due to viral mutation. More viral strains are expected to evolve in the future due to continuous virus mutations. The added selection pressure from the clinical usage of vaccines and antiviral drugs could lead to new drug resistant and immune-escape viral strains. Prior bioinformatic analysis of the SARS-CoV-2 sequences suggested that some of the C-to-U mutations may be caused by APOBEC proteins instead of the random mutations caused by viral RNA polymerase during replication or ROS^11,12,14,27,51,52^. Here we described the first experimental evidence demonstrating APOBEC enzymes, A1 + A1CF and A3A can target specific SARS-CoV-2 viral sequences for RNA editing, and the resulting mutations likely contribute to the viral replication and fitness.

We examined the C-to-U editing on selected SARS-CoV-2 RNA segments in HEK293T cells by A1 + A1CF, A3A, and A3G in an APOBEC-RNA editing assay in a cell-based system^28^ using the SSS safe sequencing approach^29,30^. The combination of the cell-based system and the SSS approach enable us to examine the individual APOBEC-mediated editing rate of specific viral RNA sequences, which could not be obtained from analyzing the currently deposited SARS-CoV-2 viral sequences that are derived as “one-consensus-sequence from one-patient”, thus, reflect only the final selected consensus viral sequences that survived the fitness selection.

The SSS approach also avoid the high-error rate associated with the conventional NGS sequencing used for obtaining the currently deposited SARS-CoV-2 viral sequences. Our results demonstrated that the three tested APOBECs show different editing rate at specific viral RNA sites, reaching as high as 22.2% for A1 + A1CF, 4.5% for A3A, and 0.18% for A3G, respectively, on the selected viral RNA transcript populations (Fig. 2). This editing efficiency at a specific site is a few orders of magnitude higher compared with the estimated ~ 10^–6^–10^–7^ random incorporation error rate for Coronaviruses replication^53^. The data here demonstrate that A1 plus its cofactor A1CF can efficiently edit specific AC motif sties of the viral RNA (Figs. 2, 3). A high mutation rate of C in AC motif was also noted in SARS-CoV-2 and Rubella Virus by prior bioinformatic analysis^3,11,27^. A3A showed a preferred dinucleotide UC motif, consistent with previous reports about the preferred motif for A3A-mediated editing of cellular RNA targets^25,54^. A3G edits a small number (n = 11) of SARS-CoV-2 RNA in our study (Fig. 2). To clearly verify A3G-mediated SARS-CoV-2 RNA editing, it is necessary to sequence more SARS-CoV-2 regions and/or study its association with certain folded RNA structures^10^.

However, the UC and AC motifs alone are not sufficient for efficient editing by A3A or A1 + A1CF. Many of the UC and AC motifs in the SARS-CoV-2 RNA showed only the background level of C-to-U mutation (Supplementary Dataset File 2), which suggest that additional RNA structural features around the UC/AC motifs should play an important role in dictating whether a UC or AC site can be efficiently edited (Fig. 3). Prior reports show that some cellular RNA targets with stem-loop structures are favored by A3A and A1 editing^25,28,34,54^. However, the exact RNA secondary/tertiary structural features that can dictate the editing efficiency of a UC/AC site in the SARS-CoV-2 genomic RNA by A3A/A1 remains to be defined. In addition, the potential changes in RNA secondary structure/stability or interactions with proteins due to the mutations caused by APOBEC-mediated editing could be interesting topics for future investigation.

A1 was not previously thought as a candidate which could edit the SARS-CoV-2 genome because of its target specificity and lack of expression in lungs, the primary target tissues of SARS-CoV-2 infection^55^. In our experimental system, the editing efficiency of A1 + A1CF on the AC motif is much higher than A3A on the UC motif (Fig. 2C,D). Interestingly, analysis of SARS-CoV-2 variants from the database also showed higher AC motif mutations (38.3%) than UC motif mutations (31.2%) (Supplementary Fig. S4), as also reported in the previous bioinformatic database analysis^3,11,27^. These results indicate that many of the AC-to-AU mutations in the SARS-CoV-2 genome from patients can be driven by A1 + A1CF mediated RNA-editing in the small intestine and liver with SARS-CoV-2 infection. Because the AC-to-AU mutations was not detected in A1 alone (Supplementary Fig. S5), it indicates that the AC-to-AU editing also requires a cofactor A1CF. Given the closely overlapping RNA target and editing efficiency by another A1-cofactor RBM47^28,32,35,50^, it’s likely that RBM47 may also enable A1 to perform AC-to-AU editing of SARS-CoV-2 RNA. Interestingly, both A1 cofactors A1CF and RBM47 were shown to physically interact with SARS-CoV-2 RNA in an interactome study^56^, offering indirect evidence that these RNA-binding A1-cofactors could recruit A1 to target SARS-CoV-2 RNA for editing in the infected tissue.

Since APOBEC proteins play an important role in immune responses against DNA and RNA viral pathogens (Refs.^19–22^ and references therein), we also investigated the possible effects of the three APOBECs on SARS-CoV-2 replication and progeny production in our experimental system (Fig. 5A). Surprisingly, expression of WT A3A in the tested cells showed significant increases of the viral RNA replication and the viral progeny production after 72 h postinfection (Fig. 5B,C, Supplementary Fig. S10), even though some increase of replication and progeny production was also observed in the inactive-A3A mutant expressing cell line (Supplementary Fig. S10). These pro-viral effect of A3A contrast to the known anti-viral effect of APOBECs^19–22^. The data indicate that the deaminase activity of A3A to mutate the viral genome plays a critical role in the pro-viral effect even though a minor deamination-independent enhancement effect could not be ruled out.

While most APOBEC-mediated C-to-U mutations detected in our assay system may be either detrimental or neutral to the virus and will be lost in the viral infection cycle, thus missing in the databank consensus viral sequence, some mutations beneficial to the virus's fitness will be selected for in the new viral strains. If so, SARS-CoV-2 can then turn the tables on the APOBEC mutational defense system for its evolution, including (but not limited to) the improvement of viral RNA replication, protein expression, evasion of host immune responses, and receptor binding and cell entry.

Even though the mechanisms by which A3A-editing promotes SARS-CoV-2 replication and progeny production may be complex and require future investigation, one of the A3A-mediated mutations present in the current circulating strains could offer insights into how SARS-CoV-2 takes advantage of A3A-mediated mutations. Among many of the detected A3A edited sites on SARS-CoV-2 RNA in our study are three C-to-U mutations located in the 5’UTR region, UC203, UC222, UC241 (Fig. 4A,B). The SARS-CoV-2 from patients detected all of these three mutations at different stages since early 2020, but only major circulating viral strains since early 2020 acquired the mutation at UC241 (Fig. 4C, Supplementary Fig. S6C), suggesting that C-to-U mutation at UC241 is selected for better viral fitness. This observation is surprising considering UC241 is in the non-coding 5′UTR region and, thus, not affecting the ORF coding of a protein, as in the case of the previously reported D614G mutation of the spike protein for viral fitness^5,38^. Therefore, this C241 to U mutation should not be related to a change of a viral protein function, such as cell surface receptor binding or polyprotein processing.

Mutations outside protein-coding ORFs of SARS-CoV-2 were previously designated as non-functional changes^3^. However, the 5′UTR region of SARS-CoV-2 has important function in regulating protein expression and viral RNA replication and virion packaging^39,40,57^. In fact, the UC241 is within the viral packaging signal sequence that has a stem-loop secondary structure which is in close proximity to the stem-loop structures involved in replication and leader transcription regulatory sequence (TRS-L)^39^ (Supplementary Fig. S14). Thus, this UC241 to UU241 mutation may impact the viral RNA packaging and virion production, and may even impact RNA replication, sub-genomic production, or/and the translation efficiency of the downstream proteins to endow the virus with better fitness. For better detection and quantification of APOBEC-mediated mutations on the viral genome over a time course of infection, the SSS sequencing method will be required. Additionally, multiple passages of the virus on cell culture or in animal models will be needed to better assess the effects of different mutations on viral fitness.

There is also the possibility of host RNA or DNA editing by WT A3A that leads to increased viral RNA replication and viral progeny production. Given the known activity of A3A in mutating both cellular genomic DNA and RNA transcripts^20,25^, the possibility of triggering certain cellular events by A3A editing/mutation activity that favors SARS-CoV-2 replication cannot be excluded and warrants future investigation.

In summary, we report here the experimental evidence demonstrating that certain sites of SARS-CoV-2 genomic RNA can be directly edited with high efficiency by A3A and A1 + A1CF to cause C-to-U mutation. Critical factors dictating the RNA-editing efficiency by these two APOBECs include a dinucleotide motif UC for A3A or AC for A1 as well as certain RNA structural features around the target C. Even though some APOBECs, including A3A, are regarded as host antiviral factors, we show here that RNA editing of SARS-CoV-2 by A3A can promote viral replication/propagation. These results suggest that SARS-CoV-2 can take advantage of APOBEC-mediated mutation for their fitness and evolution. Unlike the random mutations caused by RNA replication or ROS, the presence of the finite number of the UC/AC motifs on the SARS-CoV-2 genomic RNA and the potentially correct prediction of the viral RNA structures suggest that it is possible to predict all the possible target C sites in both the coding and non-coding regions to be edited by these APOBECs. With the new selection pressure from use of vaccines and antiviral drugs and the continued large-scale circulation of SARS-CoV-2 variants among unvaccinated and vaccinated people, such prediction may be meaningful for anticipating potential new viral mutations and the emergence immune escape and drug-resistant strains.

## Methods and materials

### The cell-based RNA editing system

The cell-based RNA editing system is adapted from previously reported in reference^28^. Briefly, reporter vectors containing DNA corresponding to the different RNA segments of SARS-CoV-2 (NC_045512.2) (see Fig. 1A,B) and the APOBEC (A1 + A1CF, A3A, and A3G) editor vectors (see Fig. 1C) were constructed. A1 + A1CF is constructed as one open reading frame (ORF) with a self-cleavage peptide T2A inserted between A1 and A1CF (A1-T2A-A1CF), which will produce individual A1 and A1CF proteins in a 1:1 ratio^28,58^. HEK293T cells were cultured in DMEM medium supplemented with 10% FBS, streptomycin (100 μg/mL), and penicillin (100 U/mL) and maintained at 37 °C, 5% CO_2_. One day before transfection, the cells (250 μL) were seeded at an approximate concentration of 250,000 cells/mL on an 8-well glass chamber (CellVis). The cells were then transfected with a mixture (25 μL) of an APOBEC editor vector (500 ng) and a SARS-CoV-2 reporter vector (50 ng) and 1.5 μL of X-tremeGENE 9 transfection reagent (Sigma) and incubated for 48 h. After harvesting the cells, RNA extraction with Trizol (Thermo Fisher) and DNA extraction with QuickExtract (EpiCentre) was performed, respectively, according to the manufacturer’s recommended instructions. The editor and reporter vectors used in this study were listed in Supplementary Dataset File 3.

### Sequencing library preparation

The extracted RNA was reverse transcribed with Accuscript High-Fidelity Reverse Transcriptase (Agilent) to produce the single-stranded cDNA using a specific primer annealing to the downstream sequence of SARS-CoV-2 reporter segments. The reaction was performed in a volume of 20 µL containing 1 µg of total RNA, 100 µM of reverse primer, 1× Accuscript buffer, 10 mM dNTP, 0.1 M DTT, 8U RNase Inhibitor, and 1 µL of Accuscript High-Fidelity Reverse Transcriptase (Agilent) for 1 h at 42 °C. The cDNA was then amplified for 2 cycles by adding a forward primer annealing to the junction region (JUNC, Fig. 1B), where the AAV intron is spliced out. In this first 2-cycle PCR amplification, the forward and reverse primers were attached to barcodes consists of 15 randomized nucleotides as the Unique Identifier (UID), plus four tri-nucleotides designating four different experimental conditions: TGA for A1 + A1CF; CAT for A3A; GTC for A3G; and ACG for Ctrl. Phusion^®^ High-Fidelity DNA Polymerase (NEB) was used for this PCR reaction: 98 °C 5 min—(98 °C 30 s, 71.4 °C 30 s, 72 °C 1 min) × 2 − 72 °C 5 min. This PCR product (330 bp) was then cleaned up using a spin column PCR cleanup kit (Thermo) to remove the free first-round barcode primers. The second-round PCR was performed for 30 cycles with Illumina flowcell adaptor primers using Phusion^®^ High-Fidelity DNA Polymerase (NEB): 98 °C 5 min—(98 °C 30 s, 72 °C 1 min) × 30 − 72 °C 5 min. All 28 (4 editors × 7 different SARS-CoV-2 substrates) of the different pooled PCR products (399 bp) were combined in equal amounts for the final libraries. The final libraries were subjected to a full HiSeq Lane (PE150, 370 M paired reads, Novogene). The primers for the sequencing library preparation were listed in Supplementary Dataset File 3.

### Analysis of safe-sequencing-system

To distinguish a true mutation from random mutation during PCR and sequencing errors, we followed the approach as reported in Ref.^29^. The details of our implementation of the method was described in Ref.^30^. We wrote Python scripts to analyze the sequencing data. We only considered those sequencing reads such that (1) at least 85% of the bases matched the reference sequence, and (2) the quality scores for all the UID bases were 30 or greater (probability of a sequencing error < 0.001). We clustered reads with the same UID and barcode into UID families. We only considered those families with at least three reads with the same UID and barcode. At each nucleotide site, the mutation frequency is calculated by dividing a numerator by a denominator. The denominator is the number of UID families that, at this particular nucleotide site, have at least three reads with quality scores of at least 20 (probability of a sequencing error < 0.01; because of this quality restriction, the denominator may be different at different sites). The numerator is the number of UID families that, at this particular site, (1) have at least three reads with quality scores of at least 20, and (2) 95% of these reads have the same base, which is different than the reference. The probability that three out of three reads will all have the same sequencing error at a site is then 10^–7^ (= (0.01^3^)/(3^2^)).

### Caco-2 stable cell line expressing APOBEC proteins

We used lentiviral transfection to construct stable Caco-2 cell lines expressing A3A, A3G, and A1 + A1CF to study the effect of APOBEC on SARS-CoV-2 replication because Caco-2 expresses the virus receptor ACE2^42^. Lentivirus was produced by lentiviral vector system pLVX-TetOne-Puro (Clon-tech) in HEK293T cells. The cells (about 2 × 10^6^ cells) were seeded in a 100 mm plate one day before transfection. The cells were then co-transfected with lentiviral packaging vectors, 1.0 μg of pdR8.91 (Gag-Pol-Tat- Rev, Addgene), 0.5 μg of pMD2.G (VSV-G, Addgene), and 1.7 μg of the pLVX-TetOne-Puro vector encoding the APOBEC proteins, using 20 μL of X-tremeGENE 9 transfection reagent (Sigma). Lentivirus-containing supernatant from infected HEK293T cells was collected after 70 h and filtered through a 0.45 μm PVDF filter (Millipore). Virions were precipitated with NaCl (0.3 M final) and PEG-6000 (8.5% final) at 4 °C for 6 h and centrifugated at 4000 rpm at 4 °C for 30 min. The pelleted virions were resuspended in 100 μL of MEM medium. Caco-2 cells (human colon epithelial cell line, ATCC) were cultured in MEM medium supplemented with 10% FBS, streptomycin (100 μg/mL), and penicillin (100 U/mL), and maintained at 37 °C, 5% CO_2_. The Caco-2 stable cell lines were generated by transducing with the lentivirus for 24 h and selected with 5 µg/mL of puromycin. The expression of A1 + A1CF, A3A, or A3G was induced by adding 1 μg/mL doxycycline for 24–96 h. Expression of these APOBEC proteins was verified by Western blot.

The ∆A3A Caco-2 cell line was created by puromycin selection after targeting N-terminus of genomic A3A exon-2 region with CRISPR-Cas9 methods (guide RNA sequence: UGGAAGCCAGCCCAGCAUCC) and inserting the SV40-promoter-Puromycin resistant gene (938 bp) through homology directed repair (HDR) system (left homology arm: 703 bp and right homology arm: 561 bp). A randomized guide RNA was used to generate a Caco-2 cell line as a negative control.

### SARS-CoV-2 virus replication and progeny production

SARS-CoV-2 propagation, infection, and viral titration were performed as previously described^59^. All SARS-CoV-2 related experiments were performed in the biosafety level 3 (BSL-3) facility (USC). For SARS-CoV-2 propagation, Vero E6-hACE2 cells were used. The cells were plated at 1.5 × 10^6^ cells in a T25 flask for 12 h and infected with SARS-CoV-2 (isolate USA-WA1/2020) at MOI 0.005 in an FBS-free DMEM medium. Virus-containing supernatant was collected when virus-induced cytopathic effect (CPE) reached approximately 80%.

To assess the effect of APOBEC (A1 + A1CF, A3A, and A3G) on SARS-CoV-2 RNA replication, the Caco-2-APOBEC stable cells (about 2 × 10^5^ cells) were plated in 12-well plates. After 15 h, cells were treated or untreated with Doxycycline for 24 h before infection. Before viral infection, the cells were washed with an FBS-free medium once. Viral infection was incubated on a rocker for 45 min at 37 °C. The cells were washed and incubated in a medium containing 10% FBS with or without Doxycycline. Total cellular RNA was extracted from the infected cells at 24, 48, 72, 96 h. Real-time quantitative PCR (qPCR) was used to quantify the viral RNA abundance level at the four different time points using viral RNA-specific primers to detect the Nsp12, S, and N regions. The qPCR of the internal actin RNA abundance level is used as a control by using actin-specific primers.

To assess the effect of APOBEC (A1 + A1CF, A3A, and A3G) on SARS-CoV-2 viral progeny production, plaque assay was used on Vero E6-hACD2 cells that has defective innate immunity and is highly sensitive to viral infection, allowing sensitive quantification of viral progeny produced from the Caco-2 cell lines. Vero E6-hACE2 cells were seeded in 12-well plates. Once cell reached confluence, cells were infected with serially diluted SARS-CoV-2 virions collected from the infected Caco-2-APOBEC stable cells that express A1 + A1CF, A3A, or A3G at 48 h and 72 h after viral infection. The medium was removed after infection, and overlay medium containing FBS-free 1 × DMEM and 1% low-melting-point agarose was added. At 48 and 72 h post-infection, cells were fixed with 4% paraformaldehyde (PFA) overnight and stained with 0.2% crystal violet. Plaques were counted on a lightbox.

### Quantitative real-time PCR

Total RNA was extracted from the SARS-CoV-2 infected Caco-2 cells using Trizol (Thermo Fisher). The extracted RNA was then reverse transcribed with the reverse primers specific to Nsp12, S, and N coding regions of SARS-CoV-2, and b-Actin as an internal control, respectively, using the high-fidelity reverse transcriptase Protoscript II (NEB). The reaction was performed in a volume of 20 µL containing 1 µg of total RNA, 100 µM reverse primer, 1× Protoscript II buffer, 10 mM dNTP, 0.1 M DTT, 8U RNase Inhibitor (40 U/µL), and 200U ProtoScript RT for 1 h at 42 °C. Quantitative real-time PCR was then performed with SYBR Green (PowerUp™ SYBR™ Green Master Mix, Thermo Fisher Scientific) in a volume of 10 µL/well containing 1 µL of reverse transcribed cDNA product from above, 0.25 µL of forward and reverse primers (10 µM), and 5 µL of PowerUp™ SYBR™ Green Master Mix (2×) using a CFX Connected Real-Time PCR machine (Bio-Rad). Primers used in this study were listed in Supplementary Dataset File 3. The indicated gene (*Nsp12*, *S*, *N*) expression levels were calculated by the 2-ΔΔCt method and normalized by b-Actin expression level.

### Western blot and antibodies

For Western blot analysis, cells were lysed in 1 × RIPA buffer (Sigma). Western blot analysis were performed from three independent transfections using FLAG-tagged APOBECs and HA-tagged A1CF. α-Tubulin: internal loading control. The lysates were then subjected to Western blot with anti-FLAG M2 mAb (F3165, Sigma, 1:3000), anti-HA mAb (HA.C5, Abcam, 1:3000), and anti-α-tubulin mAb from mouse (GT114, GeneTex, 1:5000) as primary antibodies. Cy3-labelled goat-anti-mouse mAb (PA43009, GE Healthcare, 1:3000) was subsequently used as a secondary antibody. Cy3 signals were detected and visualized using Typhoon RGB Biomolecular Imager (GE Healthcare).

### Analysis tools

The sequence logos were created by WebLogo 3 online tool with probability units (http://weblogo.threeplusone.com, ^60^). The predicted RNA secondary structures were calculated by RNAstructure^61,62^ for the local RNA region as described in Fig. 3. The number of mutational events on SARS-CoV-2 RNA segment 5′UTR-Orf1a (in Fig. 4A) was counted from the SARS-CoV-2 genome sequence data (the Nextstrain datasets from Dec. 2019 to Jan. 22nd, 2022 downloaded from the GISAID database, https://www.gisaid.org/hcov19-variants/ and https://nextstrain.org/ncov/global) using parameters set with nucleotide (x-axis) and events (y-axis). The sequencing frequency chart (in Fig. 4C) was indicated by designating the corresponding nucleotide as colored by Genotype (referred to the Nextstrain datasets: https://nextstrain.org/ncov/global, ^37^.

## Supplementary Information

Supplementary Figures.

Supplementary Information 1.

Supplementary Information 2.

Supplementary Information 3.

## Data Availability

All data is available in the manuscript or the supplementary materials. All materials used in the study are available to any researchers upon request.
