# Supplementary material for: The roles of APOBEC-mediated RNA editing in SARS-CoV-2 mutations, replication and fitness
Source: Sci Rep. 2022 Sep 13;12:14972. doi: 10.1038/s41598-022-19067-x (PMC9470679; doi:10.1038/s41598-022-19067-x)
Supplement: Supplementary file 1 — Supplementary Figures. [file 41598_2022_19067_MOESM1_ESM.pdf]

The Roles of APOBEC-mediated RNA Editing in SARS-CoV-2 Mutations, Replication and Fitness

Supplementary Materials

Kim et al.

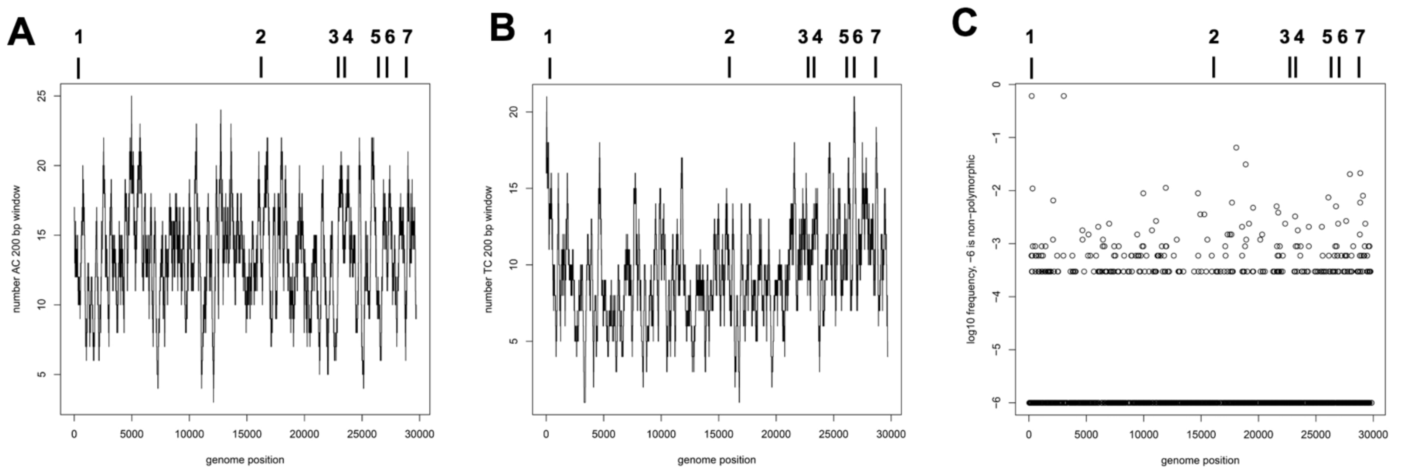

Fig. S1. Basic bioinformatic analysis to select candidates for 200nt long SARS-CoV-2 segments. Number of **(A)** AC motif and **(B)** UC motif within 200nt window across the entire SARS-CoV-2 genome. (C) The polymorphic frequency (at log10 scale) analyzed from the UCSC genome browser (https://genome.ucsc.edu/covid19.html). -6 is non-polymorphic. The positions of the seven selected segments are indicated above each panel.

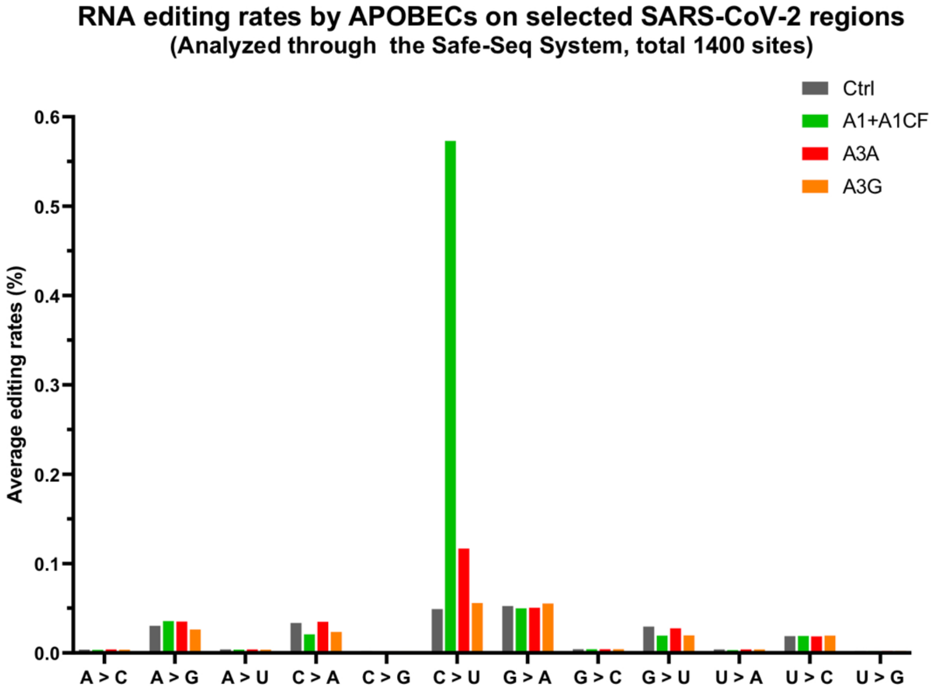

Fig. S2. The C to U RNA editing rates by APOBECs detected on the selected SARS-CoV-2 segments in our cell based assay system. Average rates (%) of all single nucleotide variations were analyzed through the Safe-Sequencing-System (SSS). See related **Supplementary Dataset File 1**.

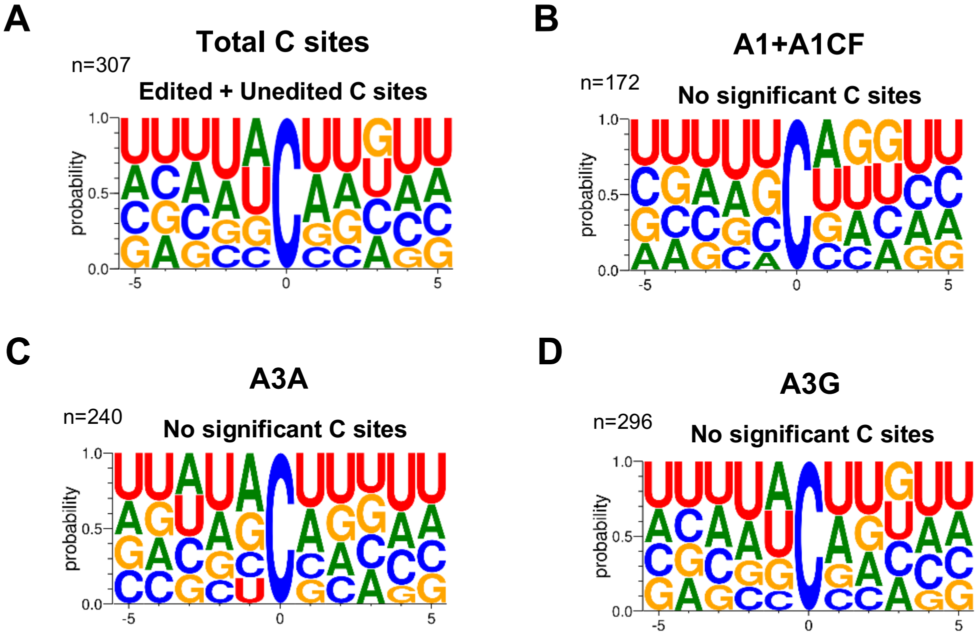

Fig. S3. Local Sequence context near all C sites and unedited C sites by corresponding APOBECs. (A) Local sequences around all C sites (± 5 nucleotides from C at position 0). **(B, C, D)** Local sequences around the unedited C sites in the presence of A1+A1CF **(B)**, A3A **(C)**, and A3G **(D)**. The unedited sites are defined as those C sites without significant editing: i.e., the C sites with 3x or less editing levels than that of Ctrl.

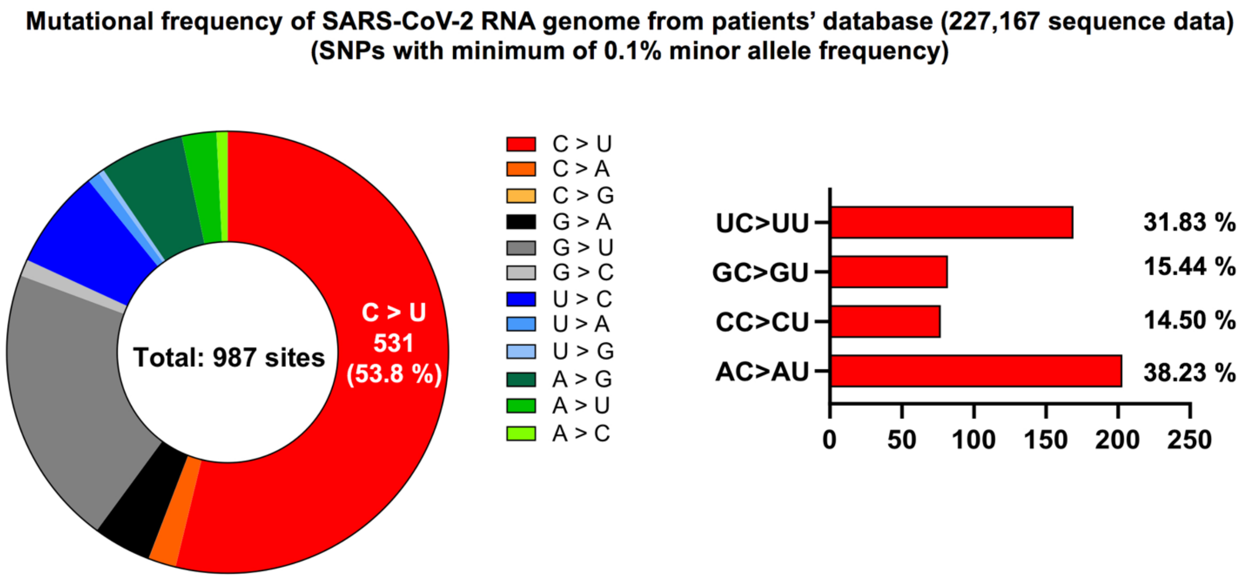

Fig. S4. Single nucleotide variations (SNPs) of the SARS-CoV-2 genome sequences database derived from patients. A total of 987 SNPs with minor allele frequencies > 0.1 % were counted from a total of 227,167 SARS-CoV-2 sequences on the UCSC genome browser (https://genome.ucsc.edu/covid19.html). The C-to-U mutation is the most common type with 53.8 % (left), of which the ratio according to the dinucleotide motifs including -1 position upstream of the mutated C are shown in the right chart.

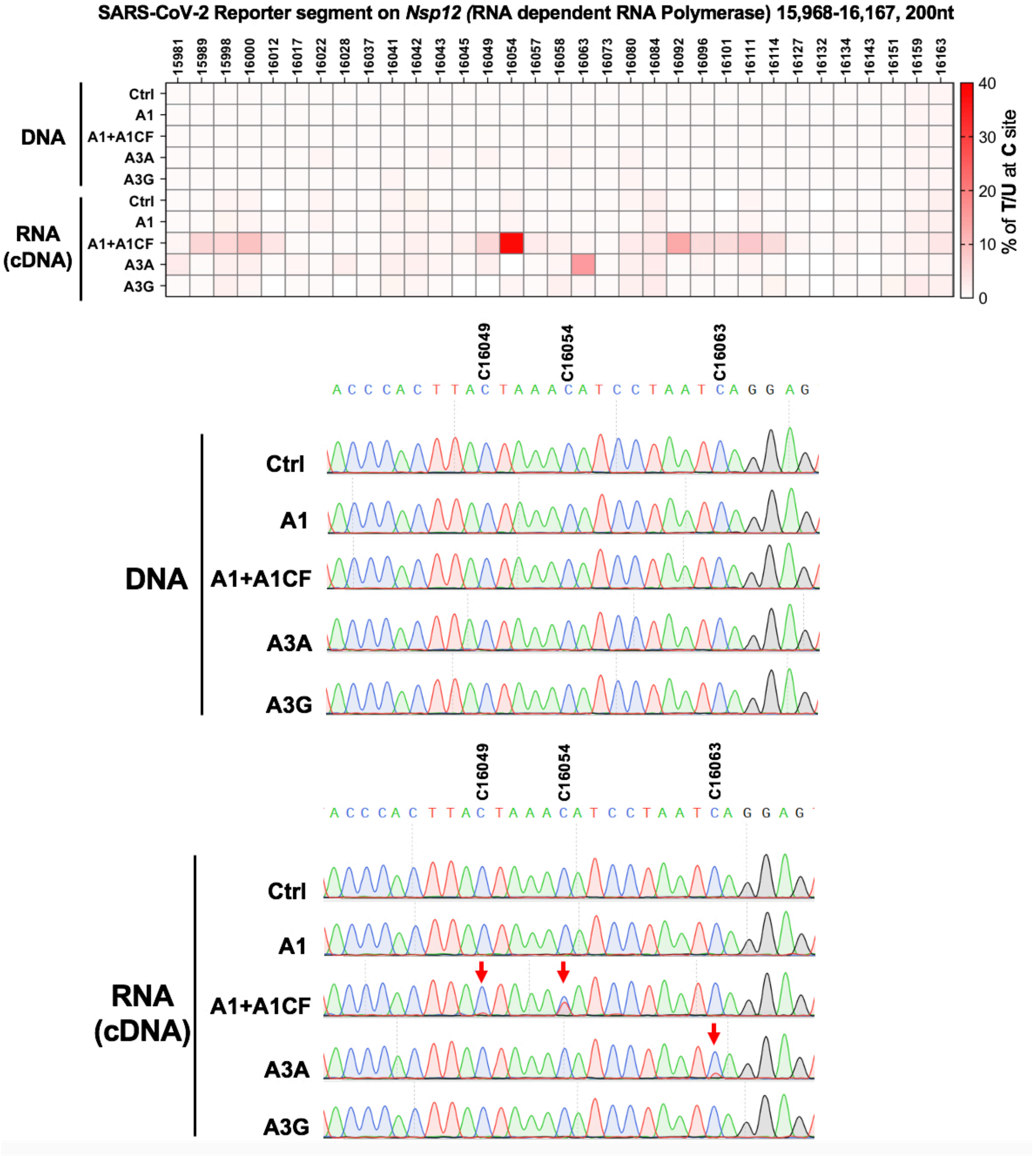

Fig. S5. Verification of C-to-U mutation as a result of direct RNA editing on the transcript of a SARS-CoV-2 reporter segment (15968-16167 nt) instead of DNA deamination on the plasmid DNA by the three APOBECs A3A, A1 (+A1CF) and A3G. The temperature-bar chart (top panel) shows the DNA and RNA C-to-T/U editing levels (%), which are based on the Sanger sequencing results of the DNA (middle panel) and the cDNA (RNA) (bottom panel). All C sites in this SARS-CoV-2 segment are marked with the virus nt sequence numbers on the top bar chart. three representative the RNA editing sites (C16049, C16054, and C16063) are indicated by red arrows.

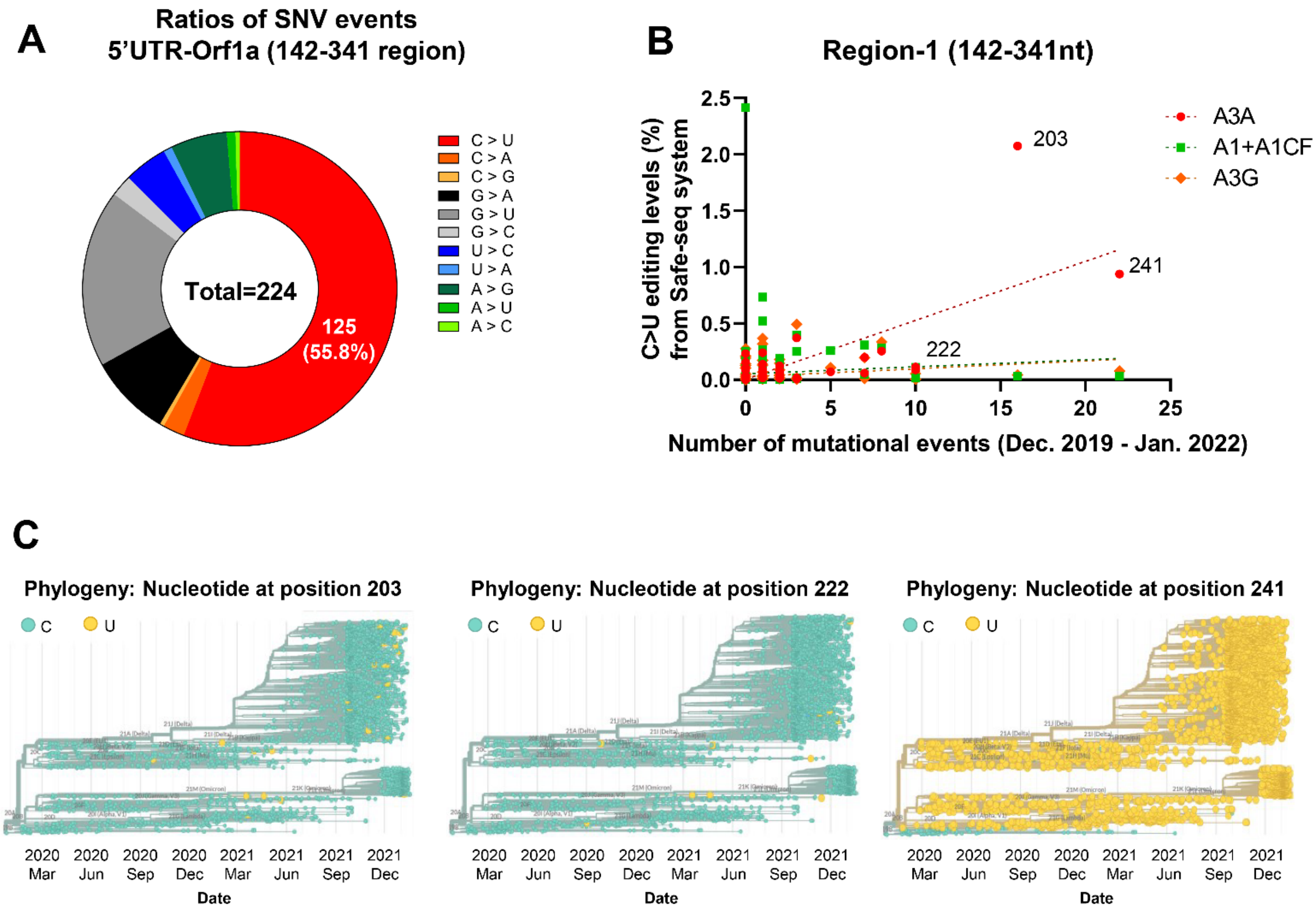

Fig. S6. Comparison of SARS-CoV-2 variants and the APOBEC-mediated RNA editing sites on the viral 5'UTR-Orf1a segment (142-341). **(A)** Ratios of all SNVs events on the 5'UTR-Orf1a segment from the sequence database (referred to the Nextstrain datasets [1]). : https://nextstrain.org/ncov/global) **(B)** Correlation of C-to-U RNA editing levels by the three APOBECs identified by our Safe-sequencing system (Y-axis) and the mutational events of SARS-CoV-2 from the sequence database between Dec. 2019 to Jan. 2022 (X-axis). Dotted lines indicate linear regressions with 95% confidence, and the case of A3A shows a positive correlation, and A1+A1CF shows a negative correlation. **(C)** Phylogenetic trees for C-to-U variant at C203, C222, and C241 (referred to the Nextstrain datasets [1]). : https://nextstrain.org/ncov/global). These phylogenetic trees correlate well with the C-to-U mutation prevalence over time at C203, C222, and C241, as shown in Fig. 4C.

A

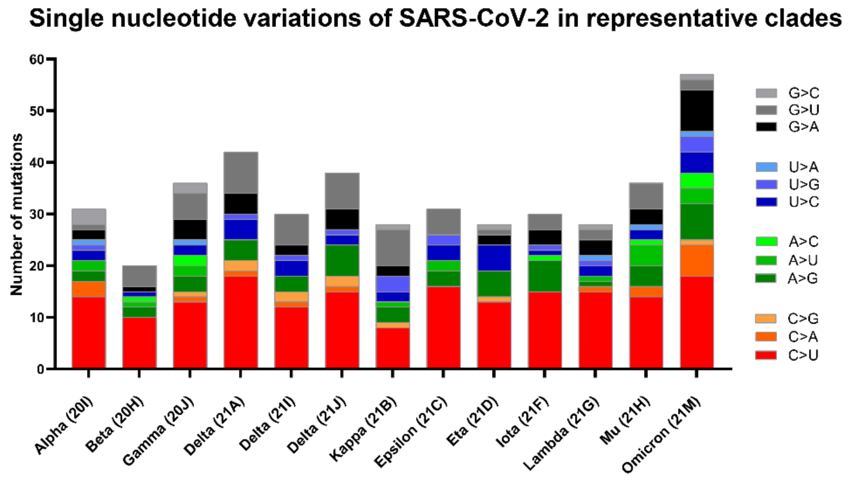

B

Mutations	Gene	Frequency (2021 Jan - Dec)	Motif	Codon
C21U	5'UTR		CC	NA
C241U	5'UTR		UC	NA
C3037U	Orf1a (Nsp3)		UC	F924F
C10029U	Orf1a (Nsp4)		AC	T3255I
C14408U	Orf1b (Nsp12)		CC	P214L
C15240U	Orf1b (Nsp12)		AC	N491N
C21762U	Spike		GC	A67V
C21846U	Spike		AC	T95I
C22674U	Spike		UC	S371F
C22686U	Spike		UC	S375F
C23525U	Spike		AC	H655Y
C24503U	Spike		CC	L981F
C25000U	Spike		AC	D1146D
C25584U	Orf3a		CC	T64T
C26270U	Envelope		AC	T9I
C27807U	Orf7/8		UC	NA
C28311U	Nucleocapsid		CC	P13L

Fig. S7. Single nucleotide variations of SARS-CoV-2 in representative clades and characterization of C-to-U mutations in the *Omicron* variant (21M). (A) Number of different single nucleotide variations (SNVs) in representative SARS-CoV-2 clades from *Alpha* (20I) to *Omicron* (21M). **(B)** Table listing the characterization of C-to-U mutations from the preferred editing motifs (UC, AC, and CC) by A3A, A1 (+A1CF), and A3G, respectively, in the representative *Omicron* variant (21M).

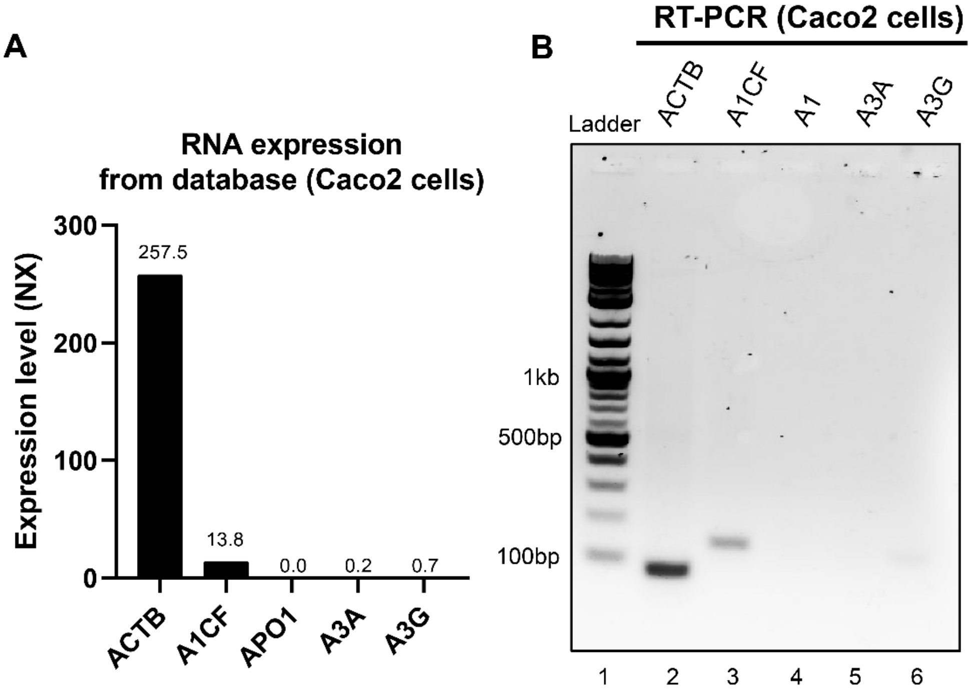

Fig. S8. Examination of endogenous RNA expression of APOBEC editors in the original **Caco-2 cells.** **(A)** Overall RNA expression levels of APOBECs (A1, A3A, and A3G) and A1CF in Caco2 cells from database. Each of gene expression values (NX) was retrieved from the human protein atlas (http://www.proteinatlas.org), which shows no or very low expression of these proteins. **(B)** RT-PCR analysis of the three APOBEC transcripts from the original Caco-2 cells. After RT of the total extracted mRNA, primers for detection of transcripts for β-actin (lane 2: 91 nt predicted), A1CF (lane 2: 139 nt predicted), A1 (lane 4: 160 nt predicted), A3A (lane 5: 143 nt predicted), and A3G (lane 6: 115 nt predicted) were used for amplification from the total cDNA. Lanes 2 was considered positive controls for genomic proteins of Caco-2 cells. This result is consistent with the analysis result from the proteinatlas database.

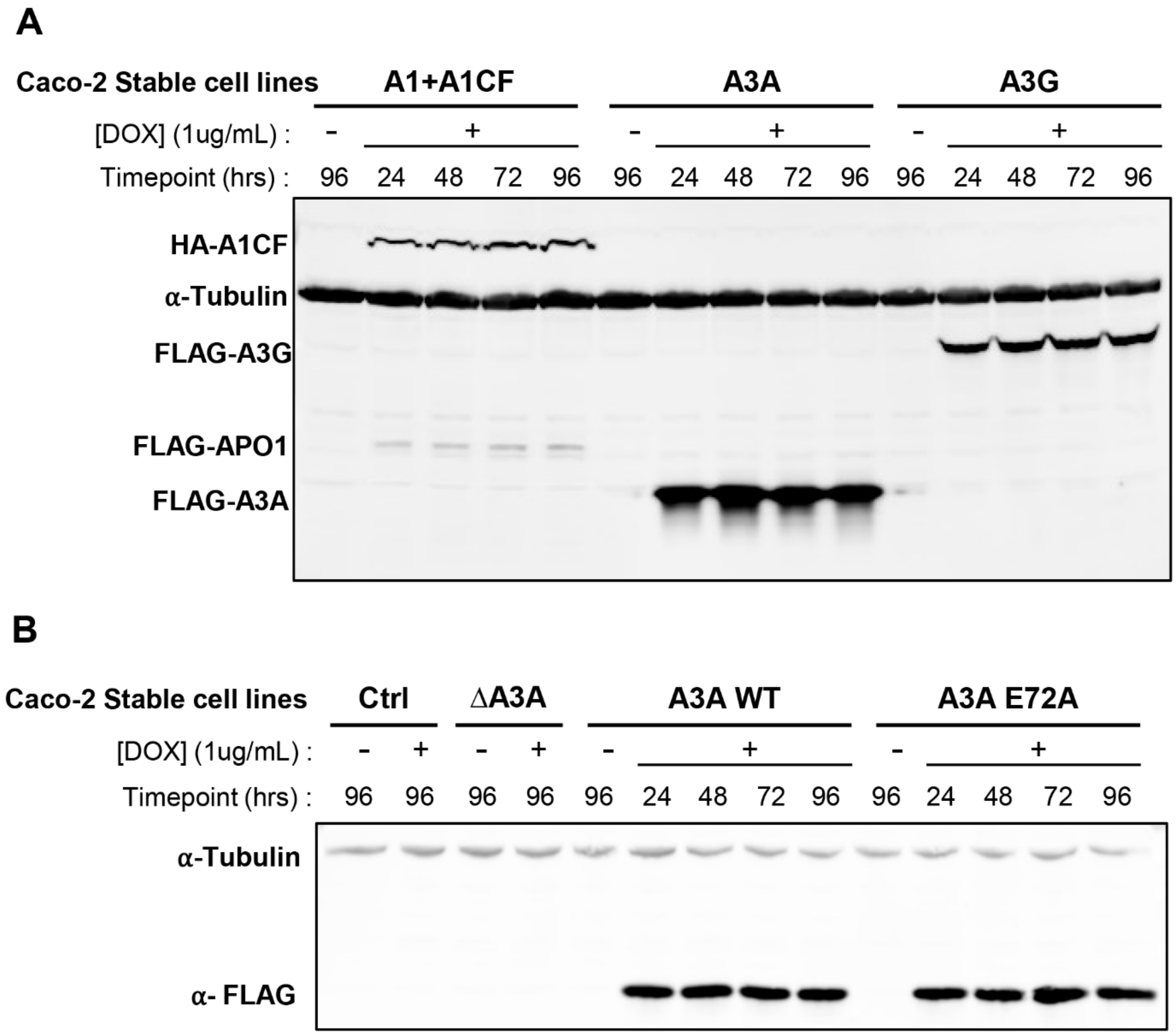

Fig. S9. Western blot analysis of APOBEC protein expression from Caco-2-APOBEC stable cell lines. **(A)** The expression level of the N-terminal FLAG-tagged A1 and HA-tagged A1CF (from A1-2A-A1CF construct), N-terminal FLAG-tagged A3A and A3G. **(B)** expression of N-terminal FLAG-tagged A3A WT and A3A E72A were detected under doxycycline treatment (1μg/mL) in different timepoints (24h, 48h, 72h, and 96h), whereas no A3A protein was detected in the A3A knockout caco-2 cell line and the control gRNA treated caco-2 cell line. α-Tubulin is the internal loading control.

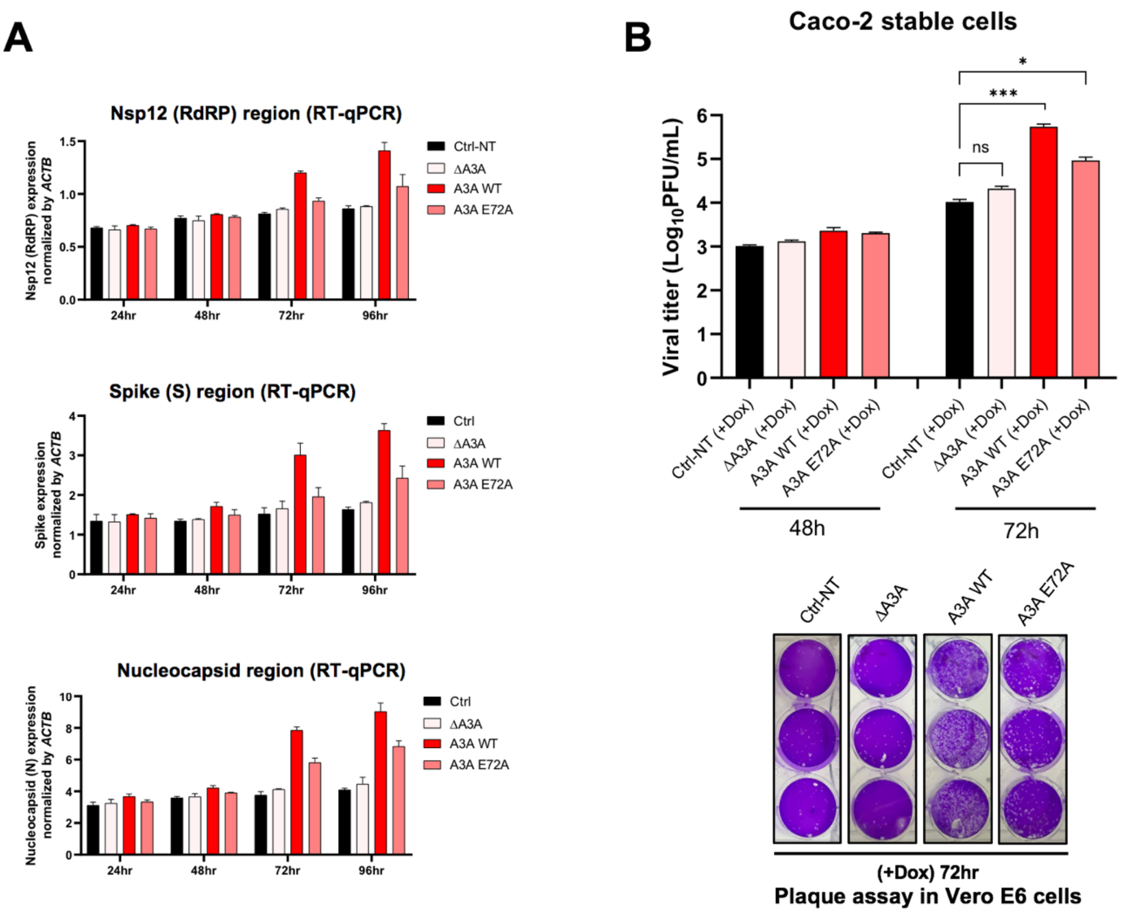

Fig. S10. SARS-CoV-2 replication and progeny production in different Coca-2 cell lines (Ctrl, ΔA3A, A3A WT, and A3A 72A)**. Ctrl:** randomized gRNA control Caco-2 cell line; **ΔA3A:** Stable Caco-2 cell line with A3A knockout by CRISPR; **A3A WT**: stable Caco-2 cell line expressing A3A wild-type protein; **A3A E72A:** stable Caco-2 cell line expressing catalytically inactive A3A mutant. **(A)** SARS-CoV-2 viral RNA replication in four different Caco-2 cell lines (Ctrl, ΔA3A, A3A WT, and A3A 72A). The viral RNA abundance was measured using real-time quantitative PCR (qPCR) to detect RNA levels by using specific primers to amplify three separate viral regions, the *Nsp12*, *S*, or *N* coding regions (see Methods). **(B)** SARS-CoV-2 progeny production in the four different Caco-2 cell lines (Ctrl, ΔA3A, A3A WT, and A3A 72A). Infectious viral progeny yield harvested in the medium at 48 hrs and 72 hrs post-infection was determined by plaque assay in Vero E6 cells (see Methods). Statistical significance was calculated by unpaired two-tailed student's t-test with *P*-values represented as: $P > 0.05$ = not significant, * = $0.01 < P < 0.05$, and *** = $P < 0.001$.

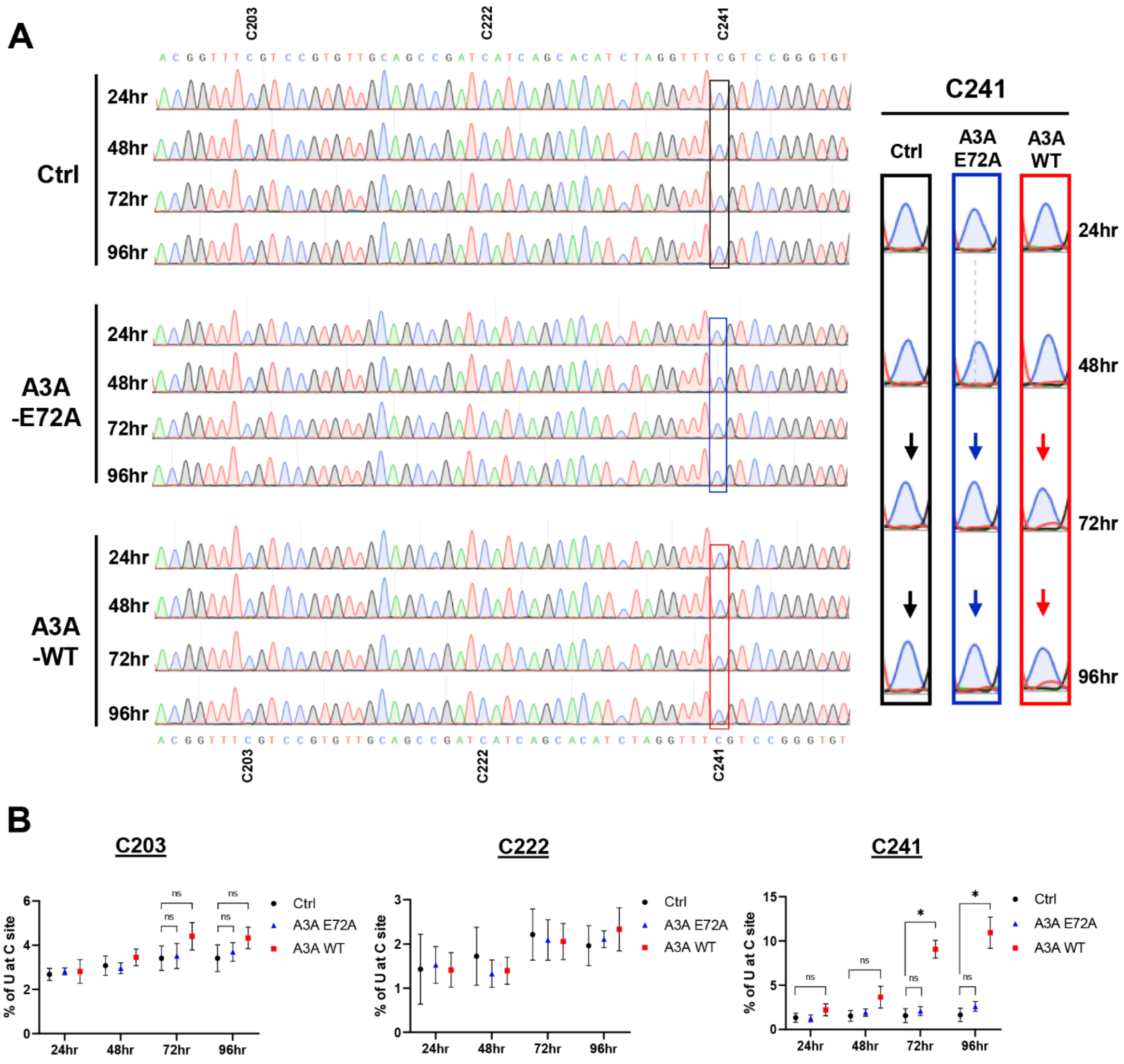

Fig. S11. Verification of C-to-U mutation caused by WT A3A-induced editing of SARS-CoV-2 virus in the 5'UTR region in Caca-2 cell culture infection assay. (A) Sanger sequencing raw chromatogram traces of the SARS-CoV-2 viral RNA around C241, C222, and C203 at different time points (24, 48, 72, and 96hr) post viral infection time in parental Caco-2 cells (Ctrl), inactive a3A mutant (A3A-E72A), and WT A3A (A3A-WT) overexpressing Caco-2 cells. The sequencing result at C241 are boxed and magnified on the right to show the appearance of T (U in RNA, red line) at C241 position only in A3A-WT starting from 72 hours post viral infection, but not in inactive A3A (A3A-E72A) and control cells without A3A. (B) Quantifying C-to-U editing levels (%) based on the Sanger sequencing results at some C sites, C203, C222, and C241. The result reveals that C241 site shows significant C-to-U editing in A3A-WT 72 and 96 hours post infection, while C203 and C222 sites show no significant C-to-U editing. Statistical significance was calculated by unpaired two-tailed student's t-test with *P*-values represented as: P > 0.05 = not significant; ns, * = 0.001 < P < 0.05.

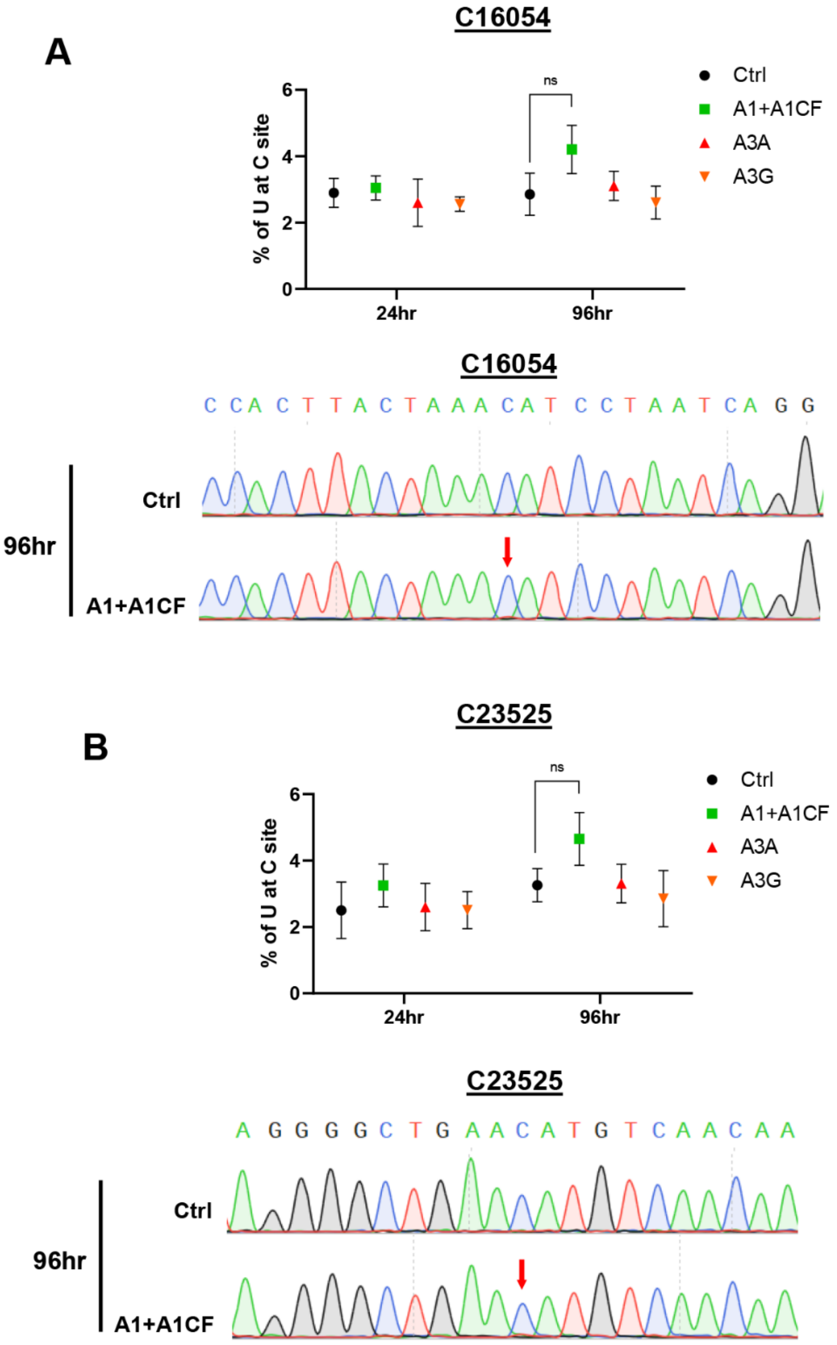

Fig. S12. Verification of C-to-U mutation of AC motifs by APOBECs in infected cells. Quantification of C-to-U editing levels (%) based on Sanger sequencing results at **(A)** C16054 and **(B)** C23525 sites. Statistical significance was calculated by unpaired two-tailed student's t-test with *P*-values represented as P > 0.05 = not significant; ns.

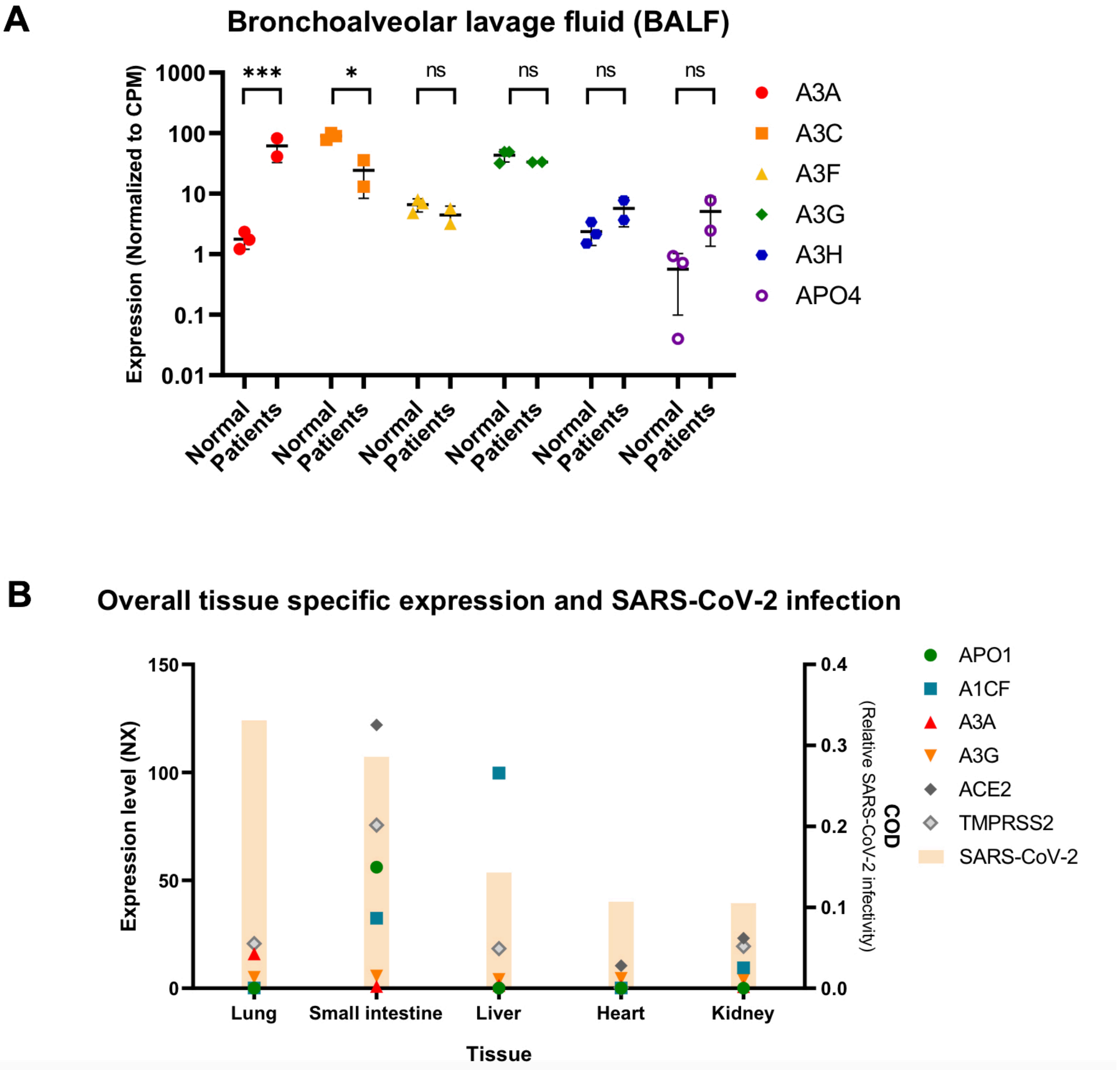

Fig. S13. Relations between SARS-CoV-2 infection and APOBEC expression. (A) Data analysis of the expression level of six APOBECs in healthy people and COVID-19 infected patients in Bronchoalveolar lavage fluid (BALF) samples (referred to the RNAseq data from reference [2]). **(B)** Overall gene expressions of the three APOBECs (A1, A3A, A3G) and A1CF in the tissues that can be infected by SARS-CoV-2. The commonness of viral detection (COD, relative SARS-CoV-2 infectivity) score for each tissue is indicated by yellow shaded boxes (referred to the COD score based on reference [3]). Each of gene expression values (NX) was retrieved from the human protein atlas (http://www.proteinatlas.org)

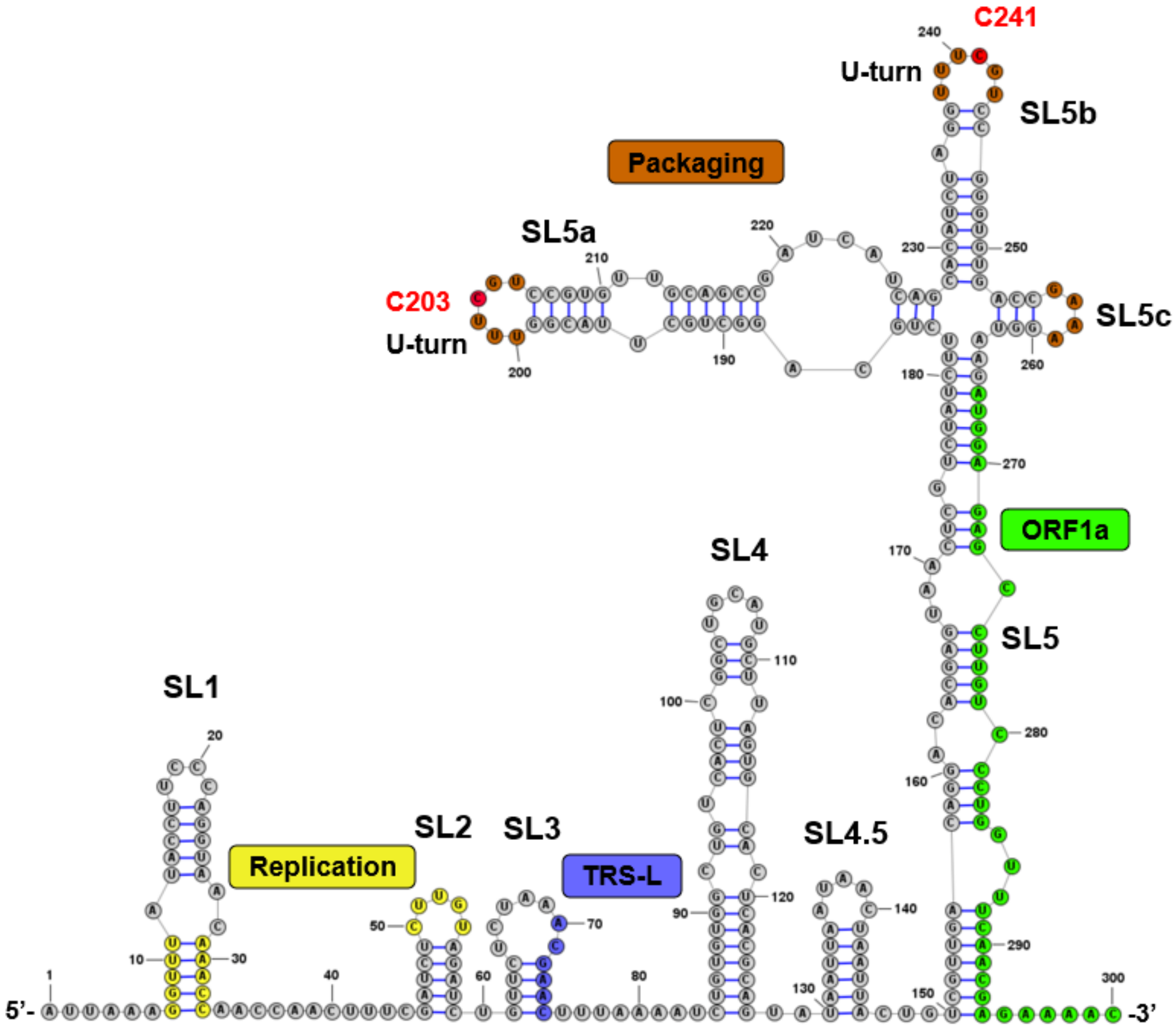

Fig. S14. A predicted secondary structure for 5'UTR region of SARS-CoV-2 and its functional motifs. The secondary structure model and functional motifs of SARS-CoV2 5'UTR were redrawn based on Miao *et. al.* [4]. The packaging signals are highlighted in brown, replication-related motifs are highlighted in yellow, the leader transcription regulatory sequence (TRS-L) shown in blue, and ORF1a (from AUG) marked in green. The A3A editing target sites U<u>C</u>241 and U<u>C</u>203 (shown in red) are located on two separate loops within the packaging signal sequences that are spaciously close to the replication related motifs and TRS-L.

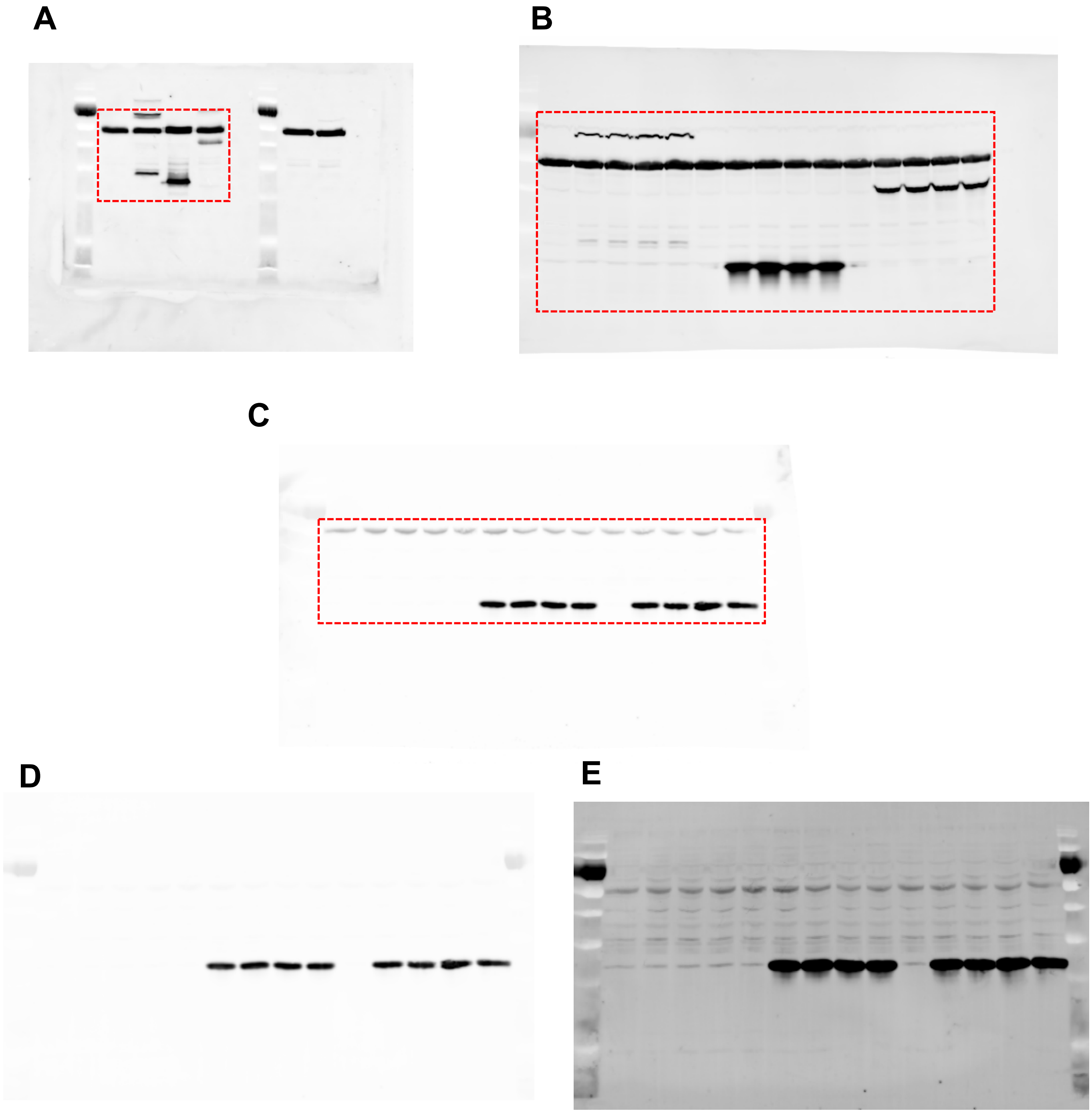

Fig. S15. Uncropped images of the western blot shown in (A) for Figure 1C, (B) for Fig. S9A, and (C) for Fig. S9B. The cropped areas are indicated with red dotted lines. For the description of each blot image, refer to the figure legends. The lower-exposed **(D)** and overexposed **(E)** images for Fig S15C.

1 Hadfield, J. *et al.* Nextstrain: real-time tracking of pathogen evolution. *Bioinformatics* **34**, 4121-4123, doi:10.1093/bioinformatics/bty407 (2018).

2 Xiong, Y. *et al.* Transcriptomic characteristics of bronchoalveolar lavage fluid and peripheral blood mononuclear cells in COVID-19 patients. *Emerg Microbes Infect* **9**, 761-770, doi:10.1080/22221751.2020.1747363 (2020).

3 Wei, Y., Silke, J. R., Aris, P. & Xia, X. Coronavirus genomes carry the signatures of their habitats. *PLoS One* **15**, e0244025, doi:10.1371/journal.pone.0244025 (2020).

4 Miao, Z., Tidu, A., Eriani, G. & Martin, F. Secondary structure of the SARS-CoV-2 5'-UTR. *RNA Biol* **18**, 447-456, doi:10.1080/15476286.2020.1814556 (2021).
